# Diagnostic accuracy of biomarkers to detect acute mesenteric ischaemia in adult patients: a systematic review and meta-analysis

**DOI:** 10.1186/s13017-023-00512-9

**Published:** 2023-09-01

**Authors:** Annika Reintam Blaser, Joel Starkopf, Martin Björck, Alastair Forbes, Karri Kase, Ele Kiisk, Kaja-Triin Laisaar, Vladislav Mihnovits, Marko Murruste, Merli Mändul, Anna-Liisa Voomets, Kadri Tamme

**Affiliations:** 1 Institute of Clinical Medicine, University of Tartu, Puusepa 8, 50406 Tartu, Estonia; 2 Department of Intensive Care Medicine, Lucerne Cantonal Hospital, Lucerne, Switzerland; 3 Department of Anaesthesiology and Intensive Care, Tartu University Hospital, Tartu, Estonia; 4 Department of Surgical Sciences, Section of Vascular Surgery, Uppsala University, Uppsala, Sweden; 5 Department of Surgery, Tartu University Hospital, Tartu, Estonia; 6 Institute of Family Medicine and Public Health, University of Tartu, Tartu, Estonia; 7 Institute of Mathematics and Statistics, University of Tartu, Tartu, Estonia; 8 Estonian Genome Center, Institute of Genomics, University of Tartu, Tartu, Estonia

**Keywords:** Acute mesenteric ischaemia, Biomarker, Diagnostic accuracy

## Abstract

**Background:**

Acute mesenteric ischaemia (AMI) is a disease with different pathophysiological mechanisms, leading to a life-threatening condition that is difficult to diagnose based solely on clinical signs. Despite widely acknowledged need for biomarkers in diagnosis of AMI, a broad systematic review on all studied biomarkers in different types of AMI is currently lacking. The aim of this study was to estimate the diagnostic accuracy of all potential biomarkers of AMI studied in humans.

**Methods:**

A systematic literature search in PubMed, The Cochrane Library, Web of Science and Scopus was conducted in December 2022. Studies assessing potential biomarkers of AMI in (at least 10) adult patients and reporting their diagnostic accuracy were included. Meta-analyses of biomarkers’ sensitivity, specificity, and positive and negative likelihood ratios were conducted. The Preferred Reporting Items for Systematic Reviews and Meta-Analyses (PRISMA) guidelines were followed, and the study quality was assessed with the QUADAS-2 tool.

**Results:**

Seventy-five studies including a total of 9914 patients assessed 18 different biomarkers in serum/plasma and one in urine (each reported in at least two studies), which were included in meta-analyses. None of the biomarkers reached a conclusive level for accurate prediction. The best predictive value overall (all studies with any type and stage of AMI pooled) was observed for Ischaemia-modified albumin (2 studies, sensitivity 94.7 and specificity 90.5), interleukin-6 (n = 4, 96.3 and 82.6), procalcitonin (n = 6, 80.1 and 86.7), and intestinal fatty acid-binding protein (I-FABP) measured in serum (n = 16, 73.9 and 90.5) or in urine (n = 4, 87.9 and 78.9). In assessment of transmural mesenteric ischaemia, urinary I-FABP (n = 2, 92.3 and 85.2) and D-dimer (n = 3, 87.6 and 83.6) showed moderate predictive value. Overall risk of bias was high, mainly because of selected study populations and unclear timings of the biomarker measurements after onset of symptoms. Combinations of biomarkers were rarely studied, not allowing meta-analyses.

**Conclusions:**

None of the studied biomarkers had sufficient sensitivity and specificity to diagnose AMI, although some biomarkers showed moderate predictive accuracy. Future studies should focus on timing of measurements of biomarkers, distinguishing between early stage and transmural necrosis, and between different types of AMI. Additionally, studies on combinations of biomarkers are warranted.

*PROSPERO registration*: CRD42022379341.

**Supplementary Information:**

The online version contains supplementary material available at 10.1186/s13017-023-00512-9.

## Background

Acute mesenteric ischemia (AMI) is a rare disease with a very high reported mortality (50–70%) showing only a modest improvement during the past few decades, with above 50% of patients still dying during the index hospitalization [1]. Such a small improvement in mortality despite widely available computed tomography, vascular surgery and interventional radiology is most likely explained by insufficient awareness and difficulties in diagnosis. AMI has different forms, which are encountered and managed by different medical specialties (e.g. emergency care physicians, vascular surgeons, interventional radiologists, visceral surgeons, gastroenterologists, intensivists), potentially complicating a uniform approach. A recent survey distributed amongst different medical specialists individually as well as in teams within different hospitals demonstrated that diagnosis of AMI is often delayed and that management is widely variable [2]. It has been shown that improved awareness (clinical suspicion) and focusing on the problem may improve outcomes [3, 4]. However, the lack of both specific symptoms and reliable biomarkers to diagnose AMI remains major factors limiting progress. Identification of reliable biomarkers is considered a priority. Previous systematic reviews assessing diagnostic accuracy of novel serum and haematological markers of AMI were published in 2017 and 2019 [5, 6]. A broad systematic review on all studied biomarkers in different types of AMI is currently lacking, and combinations of biomarkers have rarely been studied, giving a strong rationale to this study.

The aim of our study was to assess the diagnostic accuracy of all potential biomarkers for the diagnosis of AMI in adult patients. Additionally, any combinations of biomarkers that have been studied in this population were also to be assessed.

## Methods

In this systematic review and meta-analysis, we assess diagnostic accuracy of all potential biomarkers of AMI studied in adult patients. Any clinical studies including at least 10 adult patients were included, and any publications not presenting original data (e.g. reviews, editorials), case reports, cohort studies with < 10 patients, animal studies, studies in neonates and children, and studies published only as abstracts were excluded.

The population of interest was adult (> 18 years of age) patients with suspected AMI regardless of pathophysiological mechanism (occlusive arterial thrombosis or embolism, mesenteric venous thrombosis, non-occlusive mesenteric ischemia, mesenteric ischaemia due to strangulated bowel disease/obstruction—SBO).

Studies were considered eligible if:

1. A potential biomarker was measured in patients in whom AMI was suspected;
2. The diagnosis of AMI was confirmed either at surgery, CT-angiography, mesenteric angiography, endoscopy, or histopathological examination (incl. autopsy); and
3. Diagnostic accuracy of a potential biomarker was reported as sensitivity and specificity, or as true-positive (TP), true-negative (TN), false-positive (FP) and false-negative (FN) cases.

The list of pertinent biomarkers was predefined based on scoping literature searches. However, we did not exclude (studies on) other potential novel biomarkers.

### Review questions

What is the diagnostic accuracy of the following biomarkers in diagnosing AMI in adult patients?

1. Serum/plasma
2. Intestinal fatty acid-binding protein (I-FABP)
3. Alpha glutathione S transferase (alpha-GST)
4. Ischaemia-modified albumin (IMA)
5. Smooth muscle protein 22 (SM22)
6. Cobalt-albumin binding assay
7. Citrulline
8. Adropin
9. Intestinal ileal bile acid binding protein (I-BABP)
10. Hypoxia-inducible factor 1-alpha (HIF-1-alfa)
11. Fibroblast growth factor 23 (FGF-23)
12. Apelin
13. D-lactate
14. L-lactate
15. Metabolic acidosis
16. D-dimers
17. Neutrophil–lymphocyte ratio
18. Platelet–lymphocyte ratio
19. White blood cell count
20. C-reactive protein
21. Troponin
22. Creatinine
23. Urine
24. Urinary long non-coding RNA (lncRNA) H19
25. Urinary I-FABP
26. Urinary intestinal ileal bile acid binding protein (I-BABP)

What is the diagnostic accuracy of any other serum or urine biomarker in diagnosing AMI in adult patients?

What is the accuracy of any combination of biomarkers in diagnosing AMI in adult patients?

This systematic review was registered in PROSPERO registry (CRD42022379341, “Diagnostic accuracy of biomarkers to detect acute mesenteric ischaemia in adult patients: a systematic review and meta-analysis”) and performed according to the Preferred Reporting Items for Systematic Reviews and Meta-Analyses (PRISMA) guidelines.

### Searches

Literature searches were performed on 19th of December, searching PubMed, The Cochrane Library, Web of Science and Scopus since their inception until December 2022. The searches were not restricted to date or language. Additional studies were searched by screening of cross references of relevant articles, including existing systematic reviews. All search strategies are presented in Additional file 1.

### Main outcomes

- Accuracy of diagnosis of AMI
- Threshold value of positive or negative test result

### Data extraction

Titles and abstracts of studies identified utilizing the developed search strategy and from additional sources were screened independently by two reviewers to identify studies for full-text review. The selected full texts were independently assessed by two reviewers. For any disagreements during the title/abstract and full-text review, consensus was reached, involving a third reviewer if necessary. Animal, in vitro and paediatric studies, duplicates, studies that were not original or included less than 10 patients, were excluded during the title/abstract review. During the full-text review, we excluded studies that did not report biomarkers measured in blood, serum, plasma or urine; did not report extractable data of diagnostic accuracy of studied biomarkers; and studies where the reference standard for diagnosis of AMI was not applicable. Our per-protocol predefined applicable reference standards included surgery, computed tomography, angiography, endoscopy or histopathological examination. The following information was extracted independently by two reviewers from assessed full texts: study setting, patient selection, age, gender, studied biomarker and any combination of biomarkers, measurement method with reference values, timing of biomarker measurement, number of patients with AMI and without AMI, sensitivity and specificity of the biomarker for diagnosis of AMI with TP, TN, FP and FN cases, determined biomarker cut-off, diagnostic criteria used for AMI, and progression, type and localisation of AMI if available.

### Risk of bias (quality) assessment

The QUADAS-2 tool was used to assess risk of bias and applicability of included studies and completed by two research team members in parallel. Decisions were made after reaching consensus, or by involving a third reviewer if necessary. If a study was judged as “low” on all domains relating to bias/applicability, it was judged as having “low risk of bias” / “low concern regarding applicability”. If a study was judged "high" or "unclear" on one or more domains, it was judged as being “at risk of bias” / having “concerns regarding applicability”.

### Strategy for data synthesis and analysis

We constructed two-by-two contingency tables for all biomarkers. We calculated sensitivity and specificity with 95% confidence intervals (CI) based on the data (TP, TN, FP, and FN) extracted from each of the included studies. If TP, TN, FP and FN were not provided, we calculated these based on given sensitivity, specificity, sample size and AMI prevalence.

Random-effects meta-analyses were used to pool the sensitivities, specificities, positive and negative likelihood ratios in subgroups. For sensitivity and specificity analyses, we used logit-transformation in R software (V.4.1.0, R Foundation for Statistical Computing, Vienna, Austria) package meta. The confidence intervals were calculated using the Clopper–Pearson method [7].

The pooled likelihood ratios were obtained based on the bivariate model for diagnostic test accuracy in R package mada. It applies a sampling-based approach proposed by Zwinderman and Bossuyt that uses the parameters of a fit to the bivariate model to generate samples for observed sensitivities and false-positive rates [8].

The results are presented in tables with estimates and their 95% CI or in forest plots along with I^2^ statistic, τ^2^ and Cochran’s Q-test to describe the heterogeneity.

Youden index (sensitivity + specificity − 1) was used to rank the biomarkers [9].

Positive likelihood ratio > 10 and negative likelihood ratio < 0.1 were considered as high diagnostic accuracy confirming the accurate performance of a biomarker. Positive likelihood ratio > 5 and negative likelihood ratio < 0.2 were considered as moderate diagnostic accuracy showing potential for usage without being confirmative [10].

### Analysis of subgroups or subsets

We predefined the following subgroups:

1. Different types of AMI (occlusive arterial, mesenteric venous thrombosis, non-occlusive mesenteric ischaemia, mesenteric ischaemia due to strangulated bowel disease/obstruction – SBO).
2. Different progression of AMI (non-transmural / transmural intestinal ischaemia)
3. Different time points of the measurement of the biomarker (immediately at admission to hospital, perioperatively, within first 6h / 24h / > 24h of suspicion of AMI).

## Results

**Fig. 1:**
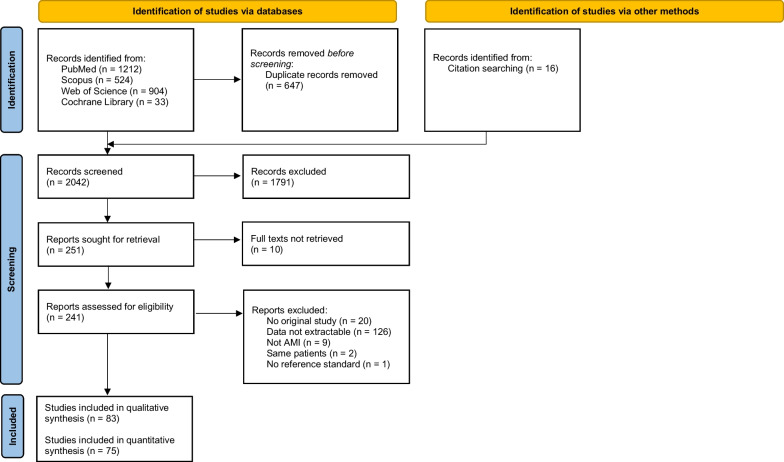
PRISMA Flow diagram

The search identified 2026 titles, and 16 additional studies. Among those, 250 studies were selected for full-text review (Fig. 1).

It was possible to extract TP, TN, FP, FN in 83 papers, and among them, 75 (with 9914 participants) provided data for quantitative analysis [11–85]. Assessment of risk of bias of all studies included in qualitative analysis is presented in Additional file 2: Table S1. All studies were judged to have some risk of bias and/or some concerns regarding applicability, and thus, it was not possible to perform the planned sensitivity analyses, excluding studies with lower quality.

It was not possible to differentiate studies/patients with early non-transmural AMI; therefore, we adapted our subgroups to “any stage” (including studies with any stage of AMI, possibly containing transmural; but excluding studies where only patients with transmural AMI were assessed) and “transmural” (including only studies on transmural AMI). Accordingly, these results need to be interpreted with caution as the proportion of “transmural” within the “any stage” is not clear.

All biomarkers included in meta-analyses with the number of studies and patients, as well as predictive values in subgroups for any stage, and “transmural” for all these biomarkers are presented in Table 1. Table 2 presents the 12 best-performing biomarkers: “overall”, “any stage” and “transmural”, ranked based on Youden index.

**Table 1:** Diagnostic accuracy of all potential biomarkers for AMI studied in meta-analyses

Biomarker	N of studies, total (incl SBO)	N of patients AMI/total	Threshold (range)	Sensitivity	Specificity	LR+	LR−
IMA							
Any stage [38, 66]	2 (1)	19/40	0.188–0.35 ABSU	94.74 (70.61;99.26)	90.48 (68.87; 97.61)	7.21 (2.28; 18.90)	0.18 (0.03; 0.48)
Transmural	0						
IL-6							
Any stage [68, 71]	2 (2)	21/31	28–40 pg/mL	100.00 (0.00;100.00)	82.25 (31.72; 97.88)	3.71 (1.42; 9.80)	0.10 (0.01; 0.42)
Transmural [73, 82]	2 (2)	23/111	40–20000 pg/mL	90.37 (64.07;98.02)	82.91 (73.98; 89.22)	4.71 (2.65; 7.99)	0.19 (0.04; 0.49)
I-FABP							
Any stage (serum/plasma) [21, 39, 45, 46, 50, 56, 58, 64, 68, 69, 72, 77]	12 (7)	299/1334	90–100000 pg/mL	73.59 (56.56;85.64)	89.79 (79.17;95.31)	4.72 (2.99; 7.26)	0.37 (0.22; 0.54)
Transmural (serum/plasma) [28, 40, 75, 79]	4 (3)	45/167	100–5787 pg/mL	76.07 (26.79;96.50)	92.05 (75.26;97.78)	5.59 (2.19; 12.60)	0.50 (0.31; 0.71)
Any stage (urine) [56, 68]	2 (1)	29/54	402–2520 pg/mL	85.96 (68.23;94.58)	72.00 (51.78;86.03)	3.19 (1.66; 6.04)	0.23 (0.07; 0.51)
Transmural (urine) [28, 75]	2 (2)	13/40	551–1000 pg/mL	92.31 (60.94;98.93)	85.22 (66.58;94.35)	5.40 (2.24; 11.70)	0.19 (0.04; 0.51)
PCT							
Any stage [51, 62, 85]	2 (1)	130/1102	2–6.6 ng/mL	79.11 (65.60;88.27)	89.12 (81.51;93.83)	7.28 (4.04; 12.30)	0.22 (0.12; 0.36)
Transmural [26, 27, 57]	3 (2)	158/285	0.25–5 ng/mL	81.92 (75.14;87.17)	80.41 (72.59;86.42)	4.10 (2.85; 5.84)	0.23 (0.16; 0.32)
Alpha-GST							
Any stage [19, 30, 35]	3 (1)	57/151	4 ng/mL	76.29 (14.96;98.33)	84.83 (76.09;90.76)	3.53 (1.16; 6.16)	0.45 (0.07; 0.97)
Transmural	0						
D-dimer							
Any stage [12, 14, 19, 20, 24, 39, 41, 43, 53, 59, 80]	11 (6)	234/1164	0.13–136 mg/L	87.92 (77.05;94.04)	69.22 (50.99; 82.94)	2.43 (1.69; 3.57)	0.26 (0.15; 0.40)
Transmural [11, 37, 83]	3 (1)	28/294	0.3–2.796 mg/L	87.56 (71.13;95.26)	83.64 (37.47;97.76)	5.78 (1.20; 23.20)	0.27 (0.10; 0.61)
CRP							
Any stage [46, 50, 55, 59, 72, 74]	6 (4)	173/940	3–232 mg/L	69.43 (31.32;91.88)	90.22 (45.58;99.03)	3.30 (1.19; 8.87)	0.60 (0.47; 0.79)
Transmural [31, 33]	2 (2)	184/377	12.6–190 mg/L	80.04 (56.67;92.47)	76.51 (53.13;90.34)	4.96 (0.85; 16.30)	0.35 (0.05; 1.17)
D-Lactate							
Any stage [19, 61, 64, 65, 72]	5 (2)	119/527	0.012–0.35 mmol/L	88.53 (70.55;96.13)	61.66 (27.32;87.31)	2.49 (1.25; 5.65)	0.23 (0.14; 0.38)
Transmural [40]	1 (0)	13/20	0.363 mmol/L	38.46 (16.98;65.64)	100.00 (0.00;100.00)	17.80 (0.63; 107.00)	0.72 (0.37; 1.36)
NLR							
Any stage [13, 16, 47, 74, 81]	5 (3)	307/692	2.55–17.9	72.62 (55.63;84.87)	80.90 (67.40;89.67)	4.59 (2.41; 8.23)	0.33 (0.20; 0.50)
Transmural [33]	1 (1)	30/129	8.0	70.00 (51.66;83.59)	23.30 (16.01;32.62)	0.91 (0.66; 1.14)	1.34 (0.64; 2.37)
L-lactate							
Any stage [15, 17, 22, 23, 25, 28, 32, 34–36, 48, 50, 53, 54, 59]	15 (12)	604/2348	2.0–5.3 mmol/l	72.99 (61.97;81.76)	69.10 (53.39;81.37)	2.21 (1.53; 3.26)	0.36 (0.33; 0.60)
Transmural [23, 28, 34, 48, 50, 63, 83]	7 (5)	205/508	2.2–4.15 mmol/l	72.96 (64.54;80.01)	77.36 (57.05; 89.79)	3.21 (1.64; 6.26)	0.39 (0.28; 0.53)
RDW							
Any stage [13, 49, 74]	3 (1)	176/472	13–14.7%	61.74 (50.64;71.74)	78.99 (64.22; 88.73)	3.23 (1.79; 5.77)	0.48 (0.37; 0.60)
Transmural	0						
LDH							
Any stage [19, 49, 50, 72]	4 (3)	118/539	147–420 U/L	78.17 (63.60;88.01)	61.42 (41.94;77.83)	2.10 (1.24; 3.69)	0.39 (0.19; 0.70)
Transmural [31, 52]	2 (2)	99/281	214–287 U/L	70.71 (61.03;78.82)	62.97 (55.72;69.67)	2.59 (1.25; 5.43)	0.44 (0.28; 0.72)
MPV							
Any stage [13, 29, 74, 76]	4 (2)	264/485	8.3–10.5 fL	66.40 (50.99;78.96)	70.51 (61.38; 78.24)	2.26 (1.34; 3.51)	0.50 (0.26; 0.80)
Transmural	0						
Citrulline							
Any stage [53, 64]	2 (1)	73/177	15.8–16.6 nmol/mL	50.68 (39.3861.92)	94.92 (46.57; 99.75)	10.30 (1.41; 42.60)	0.58 (0.43; 0.75)
Transmural	0						
WBC							
Any stage [13, 17, 18, 20, 31, 32, 35, 39, 46, 49, 50, 59, 72, 74, 81]	15 (9)	642/2107	< 4 or > 15 × 10^9^/L	69.87 (60.83; 77.59)	68.61 (52.47; 81.23)	2.08 (1.39; 3.16)	0.39 (0.34; 0.67)
Transmural [30, 33, 42, 44, 63, 67, 84]	7 (4)	219/1048	< 4 or > 15 × 10^9^/L	70.92 (58.53;80.83)	65.97 (59.55;71.84)	2.00 (1.73; 2.30)	0.47 (0.33; 0.63)
PLR							
Any stage [47]	1 (0)	125/138	250	31.20 (23.70; 9.83)	100.00 (0.00;100.00)	24.30 (0.80; 144.00)	0.78 (0.62; 1.12)
Transmural [16]	1 0)	27/168	124	75.00 (55.66;87.76)	55.50 (47.22;63.48)	1.68 (1.20; 2.18)	0.47 (0.21; 0.82)
pH							
Any stage [35, 36, 70]	3 (2)	286/1194	7.2–7.35	52.01 (16.37;85.71)	68.60 (26.21;93.08)	1.69 (1.09; 2.86)	0.71 (0.42; 0.95)
Transmural [30, 57, 79]	3 (1)	36/181	7.245–7.35	54.15 (38.98;68.58)	64.22 (55.95;71.72)	1.48 (0.99; 2.05)	0.74 (0.50; 1.00)
Bicarbonate							
Any stage	0						
Transmural [32, 44, 67]	3 (1)	85/333	18–20 mmol/L	27.38 (18.92;37.85)	87.77 (67.50;96.13)	2.73 (0.66; 8.66)	0.87 (0.69; 1.22)

Alpha-GST—alpha glutathione S transferase (alpha-GST); AMI—acute mesenteric ischaemia; CRP—C-reactive protein; I-FABP—intestinal fatty acid-binding protein; IL-6—interleukin 6; IMA—ischaemia-modified albumin; LDH—lactate dehydrogenase; LR + – positive likelihood ratio; LR-—negative likelihood ratio; MPV—mean platelet volume; NLR—neutrophil–lymphocyte ratio; NOMI—non-occlusive mesenteric ischaemia; PCT—procalcitonin; PLR—platelet–lymphocyte ratio; RDW—red cell distribution width; WBC—white blood cell count; SBO—strangulated bowel disease

“Including SBO”—studies assessing only SBO and studies assessing any type of AMI stating including SBO or not stating excluding it

Any stage—studies assessing different stages of AMI, including but not limited to non-transmural and transmural; Transmural—studies assessing transmural AMI, with control group including non-transmural AMI

Biomarkers are presented in the order based on Youden index (highest to lowest) in the analysis including all available studies

**Table 2:** Ranking of twelve best biomarkers according to Youden index

	Any stage		Transmural		Overall	
	Biomarker	Youden index	Biomarker	Youden index	Biomarker	Youden index
1	IMA	0.85	I-FABP urine	0.77	IMA *	0.85
2	IL-6	0.82	IL-6	0.73	IL-6 ^#^	0.79
3	PCT	0.68	D-dimer	0.71	I-FABP urine	0.67
4	I-FABP serum	0.63	I-FABP serum	0.68	PCT	0.67
5	alpha-GST	0.61	PCT	0.62	I-FABP serum	0.64
6	CRP	0.60	CRP	0.56	alpha-GST *	0.61
7	I-FABP urine	0.58	L-lactate	0.50	D-dimer	0.60
8	D-dimer	0.57	D-lactate	0.38	CRP	0.58
9	NLR	0.54	WBC	0.37	D-lactate	0.55
10	D-lactate	0.50	LDH	0.34	NLR	0.47
11	Citrulline	0.46	PLR	0.31	Citrulline *	0.46
12	L-lactate	0.42	pH	0.18	L-lactate	0.44

Alpha-GST—alpha glutathione S transferase (alpha-GST); CRP—C-reactive protein; I-FABP—intestinal fatty acid-binding protein; IL-6—interleukin 6; IMA—ischaemia-modified albumin; LDH—lactate dehydrogenase; NLR—neutrophil–lymphocyte ratio; PCT—procalcitonin; PLR—platelet–lymphocyte ratio; WBC—white blood cell count

Any stage—studies assessing different stages of AMI, including but not limited to non-transmural and transmural; Transmural—studies assessing transmural AMI, with control group including non-transmural AMI; Overall—all studies pooled independent of stage and type of AMI

*No study on transmural acute mesenteric ischaemia of any type

^#^No study on non-occlusive mesenteric ischaemia

Forest plots for analysed biomarkers are presented in Figs. 2–5 (analyses including > 10 studies) and in Additional file 3: Figures S1–S15 (analyses including < 10 studies). Next to overall diagnostic accuracy (pooling all studies on a specific biomarker), we present studies assessing only non-occlusive mesenteric ischaemia (NOMI) or ischaemia due to strangulating bowel disease (SBO) separately on these figures, where applicable.

**Fig. 2:**
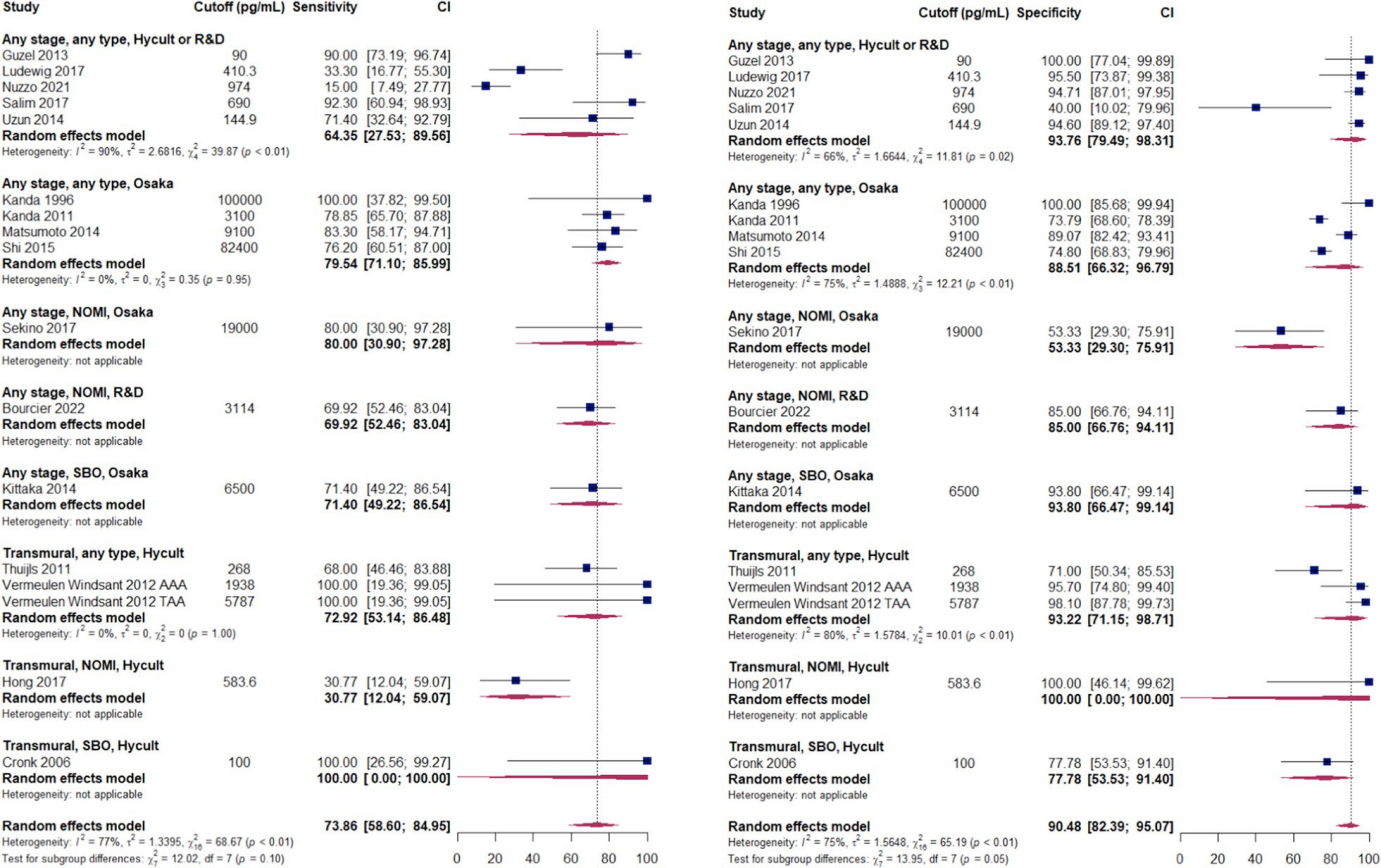
Sensitivity (panel A) and specificity (panel B) of serum intestinal fatty acid-binding protein (I-FABP) predicting AMI. AMI—acute mesenteric ischaemia; NOMI—non-occlusive mesenteric ischaemia; SBO—strangulated bowel disease. Any stage—studies assessing different stages of AMI, including but not limited to non-transmural and transmural; Transmural—studies assessing transmural AMI, with control group including non-transmural AMI. Comment: Uzun 2014 included healthy volunteers as control. Hycult—Hycult Biotech measurement kit from Uden, the Netherlands. Osaka—D.S. Pharma Biomedical measurement kit from Osaka, Japan. R&D—R&D Systems measurement kit from Minneapolis, USA

For most of the biomarkers, different thresholds/cut-offs were used in individual studies, making interpretation of results difficult. It was not possible to analyse biomarkers separately in vascular AMI and SBO, because most of the studies either included SBO under the broad group of “any type of AMI” (see Table 1) or did not specify the exclusion of SBO.

### Diagnostic accuracy of the biomarkers

None of the studied biomarkers demonstrated high diagnostic accuracy, whereas a few showed modest diagnostic accuracy (Table 1).

**Fig. 3:**
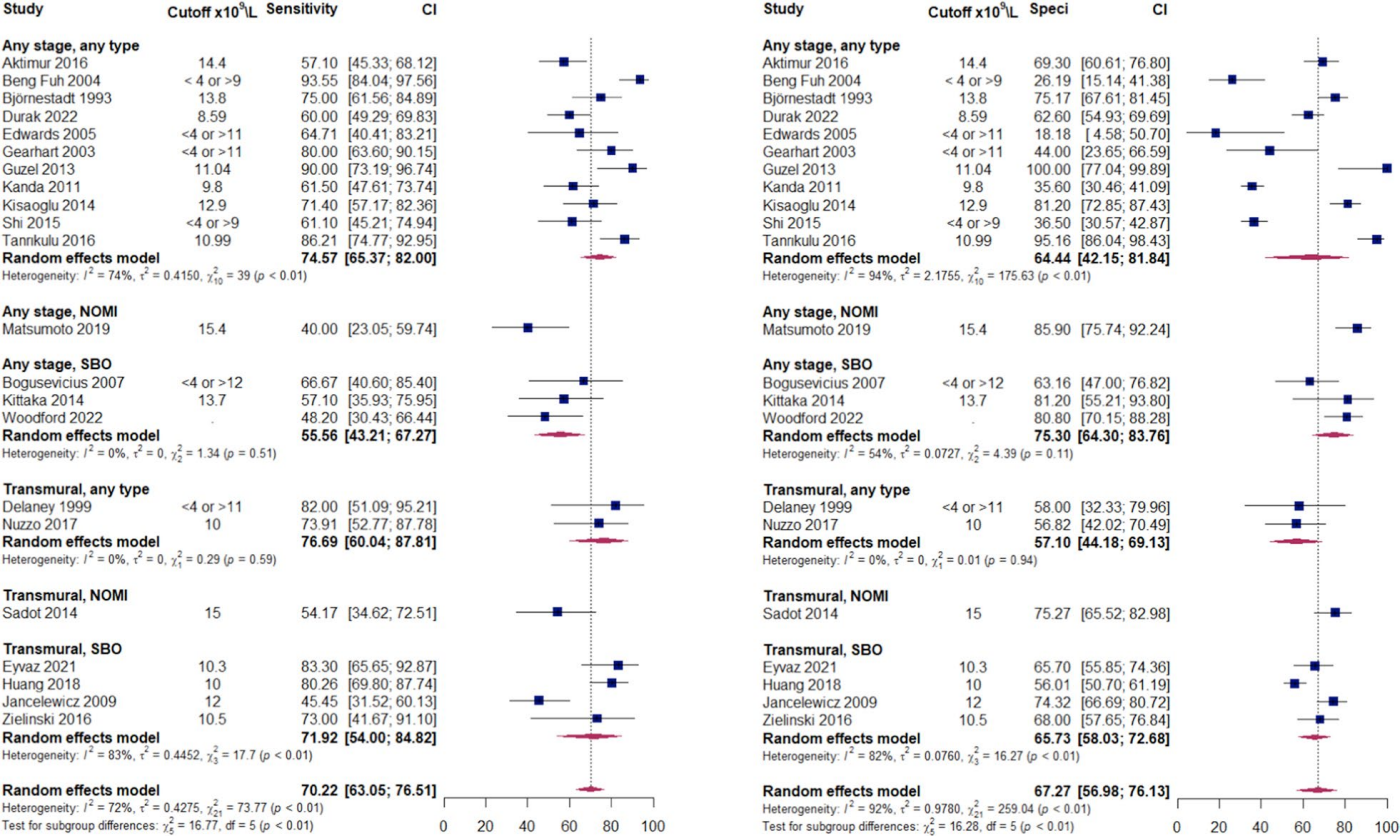
Sensitivity (panel A) and specificity (panel B) of white blood cell count (WBC) predicting AMI. AMI—acute mesenteric ischaemia; NOMI—non-occlusive mesenteric ischaemia; SBO—strangulated bowel disease. Any stage—studies assessing different stages of AMI, including but not limited to non-transmural and transmural; Transmural—studies assessing transmural AMI, with control group including non-transmural AMI

The inflammatory markers demonstrated relatively high predictive values (Tables 1 and 2), with IL-6 showing the best prediction. Figure 3 provides an overview of the performance of the white blood cell count—as a more commonly used inflammatory marker, while other inflammatory markers are presented in Additional file 3: Figures S2–S4.

**Fig. 4:**
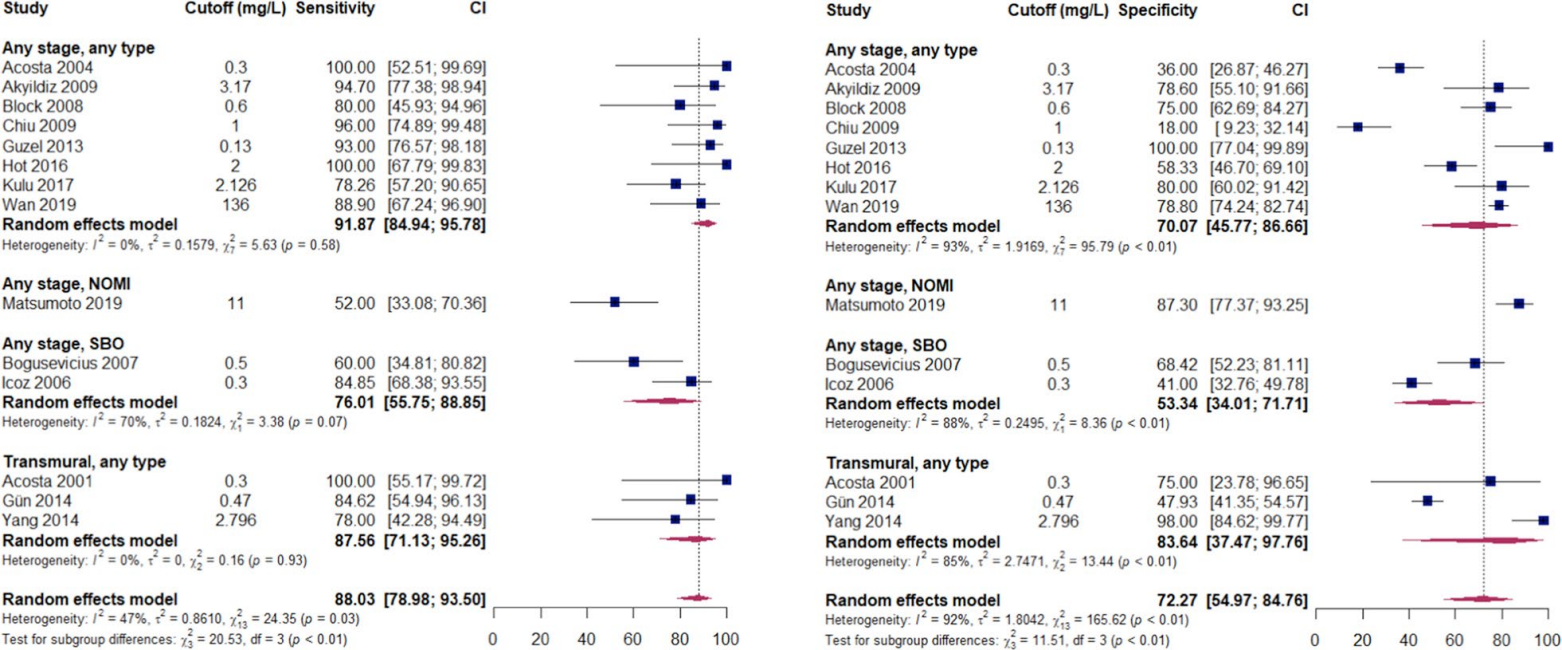
Sensitivity (panel A) and specificity (panel B) of serum D-dimers predicting AMI. AMI—acute mesenteric ischaemia; NOMI—non-occlusive mesenteric ischaemia; SBO—strangulated bowel disease. Any stage—studies assessing different stages of AMI, including but not limited to non-transmural and transmural; Transmural—studies assessing transmural AMI, with control group including non-transmural AMI

Measurement of D-dimers had insufficient predictive value for AMI at any stage but performed better in studies assessing transmural ischaemia (Fig. 4 and Table 1). Heterogeneous cut-offs complicate the interpretation of results, but it appears that patients with AMI do not present with normal values of D-dimers.

I-FABP (Fig. 2), the most studied novel biomarker, reached moderate diagnostic accuracy, although several recent studies showed rather disappointing results [56, 64]. Interpretation of data for this biomarker is further complicated by the multiple methods of laboratory analytics as well as highly variable thresholds for abnormality. Our analysis suggests that urinary I-FABP may perform better for transmural AMI (Additional file 3: Figure S1 and Tables 1 and 2), but this result is based on only two studies.

**Fig. 5:**
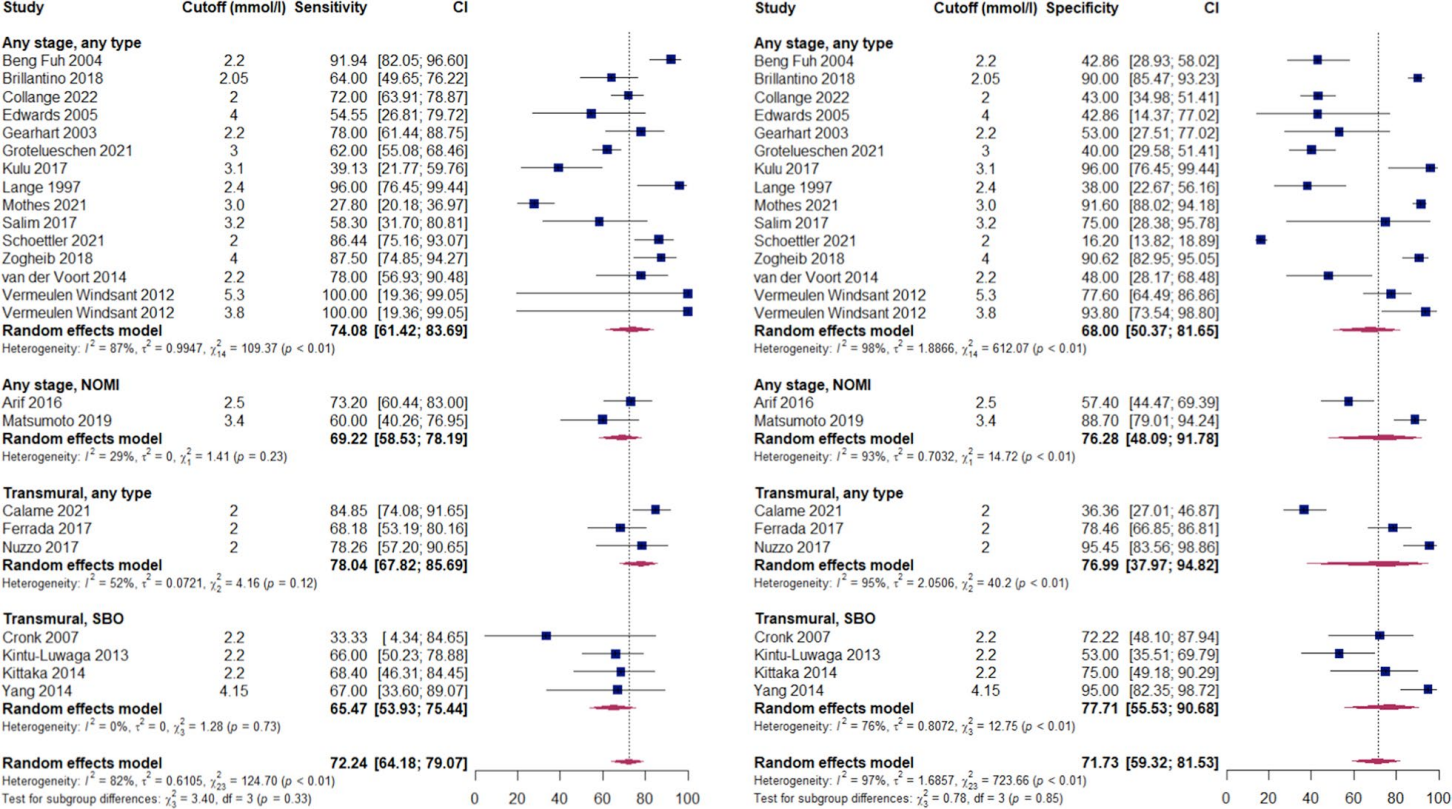
Sensitivity (panel A) and specificity (panel B) of blood L-lactate predicting AMI. AMI—acute mesenteric ischaemia; NOMI—non-occlusive mesenteric ischaemia; SBO—strangulated bowel disease. Any stage—studies assessing different stages of AMI, including but not limited to non-transmural and transmural; Transmural—studies assessing transmural AMI, with control group including non-transmural AMI

L-lactate (Fig. 5), probably the most studied biomarker of AMI, did not show sufficient diagnostic accuracy in our analysis and should not be considered an early biomarker of AMI. Some additional value of this biomarker in diagnosing transmural AMI is not excluded, because only inflammatory markers that are also not specific, and I-FABP which is not promptly available in clinical practice, performed better in our analysis (Table 2).

For a number of biomarkers, the sensitivity and specificity were reported (or could be calculated) in only one study and meta-analysis was not possible. These biomarkers were:

Stromal cell-derived factor-1 (SDF-1) (sensitivity and specificity 91 and 95%, respectively) [86].

Serum long-coding RNA H19 (94 and 100%) [87].

Serum IL-8 (88 and 100%) [71].

Serum creatine kinase BB isoenzyme (CK-BB) (63 and 100%) [88].

Plasma presepsin (89 and 85%) [89].

Serum creatinine with a cut-off of 200 micromol/L (58 and 97%) [90].

Serum L-FABP (59 and 88%, respectively) [75].

Serum hypoxia-induced factor alpha (HIF1-α) (75 and 70%) [91].

Serum smooth muscle actin (54 and 100%) [40].

Serum endothelin-1 (51 and 94%) [92].

Serum adropin (65 and 70%) [91].

Serum I-BABP (64 and 63%) [75].

Cell-free plasma DNA (54 and 84%) [93].

Urinary long-coding RNA H19 (80 and 100%) [87].

Urinary I-BABP (70 and 89%) [75].

Urinary L-FABP (80 and 78%) [75].

Other biomarkers assessed in individual studies as potential biomarkers of AMI are not presented as they were not considered novel and had been excluded from our predefined list of interest. These were haemoglobin, haematocrit, erythrocyte volume fraction, immature granulocytes, delta neutrophil index, fibrinogen, prothrombin, blood urea nitrogen, creatine phosphokinase, amylase, ASAT, ALAT and phosphate.

It was not possible to perform subgroup analyses based on timing of biomarker measurements. The time elapsed from the onset of symptoms until biomarker measurement was generally not reported in studies, and the exact times after hospital admission also remained unclear.

Scores/combinations of biomarkers were assessed in only 5 studies [23, 35, 42, 85, 94], mainly combining laboratory biomarkers with radiological or clinical markers and thus not permitting any meta-analysis.

## Discussion

In this systematic review, a considerable number of studies assessing biomarkers of AMI were identified. Despite this increasing body of evidence, no biomarker currently provides sufficient diagnostic accuracy to be recommended for clinical use. Available evidence is hard to interpret due to:

- Different cut-offs and laboratory methods in different studies;
- A lack of differentiation between different stages of AMI (non-transmural vs transmural) in most of the studies;
- A lack of differentiation between different types of AMI in most of the studies;
- Missing data on timing of biomarker measurement after development of symptoms.

Compared to previous systematic reviews, more studies on existing and novel biomarkers were included. No breakthrough in defining a reliable biomarker with acceptable sensitivity and specificity was observed however [5, 6]. Novel biomarkers such as IMA and alpha glutathione S transferase (alpha-GST) were associated with great hope a few years ago, but no newer studies were identified than those in the systematic review by Treskes in 2017 [5]. Newer studies assessing I-FABP have been published, but do not confirm the initial enthusiasm [21, 40, 56, 64, 68]. Accordingly, a moderate diagnostic accuracy may be considered disappointing for I-FABP, as the hope was that I-FABP was specific and would provide good diagnostic accuracy [5, 95–97]. However, the diagnostic value of I-FABP may be dependent on timing of its measurement [56].

Although nonspecific, inflammatory biomarkers such as IL-6, CRP and PCT performed relatively well in our analysis when compared to the novel and supposedly specific biomarkers, probably because of systemic inflammation from ischaemic injury to the bowel occurring in the later stages of AMI. Our analyses support this rationale, showing that inflammatory biomarkers may perform better in predicting transmural AMI. At the same time, inflammatory biomarkers may not be able to distinguish between severe inflammation in the bowel/peritoneal cavity of other causes vs. mesenteric ischaemia [71]. Ideally, a biomarker should be specific and diagnostic in the early phases of AMI, to enable salvage of the threatened bowel and these criteria are probably not fulfilled with inflammatory markers. Additionally, it is difficult to interpret inflammatory markers in patients with NOMI who usually have an active inflammatory state due to their severe underlying illness and its treatment (e.g. ICU patients) [98, 99]. Of note, there was no study assessing IL-6 in NOMI.

Lactate is often used in clinical practice today; however, it clearly should not be used for exclusion of AMI [100]. Lactate can be effectively metabolized in the liver, explaining why it does not serve as an early marker of AMI. Increased metabolism may cover increased production, whereas decreased metabolism may lead to elevated values without a relevant increase in production [101, 102]. However, elevated lactate should still call for our attention and maybe trigger further investigation in patients with suspected AMI [103].

As one biomarker is currently insufficient to diagnose AMI, possible combinations of different biomarkers should be studied hoping for an additive value in diagnosis. At the same time, a rapid turn-round in laboratory analytics is an important factor necessary for any future biomarker of AMI.

### Strengths and limitations

The main strength of our study is the updated synthesis of evidence on diagnostic accuracy of the potential biomarkers of AMI. To the best of our knowledge, it is the first systematic review attempting separation of transmural ischaemia from earlier stages of AMI.

The limitations of our study are mainly related to the original studies that are heterogeneous regarding patient populations (incl. control groups), types of AMI, laboratory methods and cut-offs of biomarkers and often do not report the time from development of symptoms to measurement of biomarkers. Thus, all the studies in our review were judged as being at risk of bias and/or having concerns regarding applicability. However, uncovering the need to set certain methodological standards for studies on AMI biomarkers could also be considered a strength of our study.

Additionally, we were not able to clearly separate non-transmural from transmural AMI and vascular AMI from SBO in our analyses.

## Conclusions

Currently, based on available evidence, no single biomarker enables accurate diagnosis of AMI, whereas combinations of these biomarkers have rarely been studied. Available evidence carries considerable risk of bias, is very heterogeneous and does not allow precise distinctions between different types and stages of AMI. Inflammatory markers and D-dimers may be considered to assist in diagnosis of transmural ischaemia. Future studies should focus on timing of measurements of biomarkers, considering different biomarkers for diagnosis of early stage of AMI and transmural necrosis.

### Supplementary Information

**Additional file 1:** Search strategies.

**Additional file 2: Table S1.** Risk of bias assessment.

**Additional file 3: Figures S1-S15.** Forest plots.

## Data Availability

Template data collection forms and data used for analyses can be made available on request.

## Abbreviations

ABSU: Absorbance units
ALAT: Alanine transaminase
Alpha-GST: Alpha glutathione S transferase
AMI: Acute mesenteric ischemia
ASAT: Aspartate aminotransferase
BE: Base excess
CABA: Cobalt-albumin binding assay
CI: Confidence interval
CK-BB: Serum creatine kinase BB isoenzyme
CRP: C-reactive protein
FGF-23: Fibroblast growth factor 23
FN: False negative
FP: False positive
HIF1-α: Hypoxia-inducible factor 1-alpha
I-BABP: Intestinal ileal bile acid binding protein
ICU: Intensive care unit
I-FABP: Intestinal fatty-acid-binding protein
IL-6: Interleukin 6
IL-8: Interleukin 8
IMA: Ischaemia-modified albumin
IQR: Interquartile range
LDH: Lactate dehydrogenase
L-FABP: Liver fatty acid-binding protein
RDW: Red cell distribution width
lncRNA H19: Long non-coding RNA
LR-: Negative likelihood ratio
LR+: Positive likelihood ratio
MPV: Mean platelet volume
NLR: Neutrophil–lymphocyte ratio
NOMI: Non-occlusive mesenteric ischemia
NPV: Negative predictive value
PCT: Procalcitonin
PLR: Platelet–lymphocyte ratio
PPV: Positive predictive value
SBO: Strangulated bowel disease/obstruction
SDF-1: Stromal cell-derived factor-1
SM22: Smooth muscle protein 22
TN: True negative
TP: True positive
WBC: White blood cells count
