# Supplementary material for: Diagnostic accuracy of biomarkers to detect acute mesenteric ischaemia in adult patients: a systematic review and meta-analysis
Source: World J Emerg Surg. 2023 Sep 1;18:44. doi: 10.1186/s13017-023-00512-9 (PMC10474684; doi:10.1186/s13017-023-00512-9)
Supplement: Supplementary file 1 — Additional file 1: Search strategies. [file 13017_2023_512_MOESM1_ESM.docx]

Supplement 1. Search strategies

**PubMed** 19 December 2022

((((((intestinal[Title/Abstract] OR intestine[Title/Abstract] OR mesenteric[Title/Abstract] OR mesentery[Title/Abstract] OR bowel[Title/Abstract] OR gut[Title/Abstract] OR enteric[Title/Abstract] OR enteral[Title/Abstract] OR "small bowel"[Title/Abstract] OR small-bowel[Title/Abstract] OR "large bowel"[Title/Abstract] OR large-bowel[Title/Abstract] OR colon[Title/Abstract]) AND (ischemia[Title/Abstract] OR ischaemia[Title/Abstract] OR ishemia[Title/Abstract] OR ishaemia[Title/Abstract] OR ishemic[Title/Abstract] OR ishaemic[Title/Abstract] OR ischemic[Title/Abstract] OR ischaemic[Title/Abstract] OR necrosis[Title/Abstract] OR necrotic[Title/Abstract] OR gangrene[Title/Abstract] OR gangrenous[Title/Abstract] OR infarction[Title/Abstract] OR infarcted[Title/Abstract])) OR (abdominal compartment syndrome[Title/Abstract] OR mesenteric arterial thrombosis[Title/Abstract] OR mesenteric arterial embolism[Title/Abstract] OR mesenteric embolus[Title/Abstract] OR mesenteric thrombus[Title/Abstract] OR mesenteric thromboembolism[Title/Abstract] OR mesenteric venous thrombosis[Title/Abstract] OR non-occlusive intestinal ischemia[Title/Abstract] OR nonocclusive intestinal ischemia[Title/Abstract] OR non-occlusive intestinal ischaemia[Title/Abstract] OR nonocclusive intestinal ischaemia[Title/Abstract] OR non-occlusive mesenteric ischemia[Title/Abstract] OR nonocclusive mesenteric ischemia[Title/Abstract] OR non-occlusive mesenteric ischaemia[Title/Abstract] OR nonocclusive mesenteric ischaemia[Title/Abstract] OR mesenteric vascular insufficiency[Title/Abstract] OR vascular insufficiency of intestine[Title/Abstract] OR intestinal vascular insuffuciency[Title/Abstract] OR mesenteric infarction[Title/Abstract] OR ischemic colitis[Title/Abstract] OR ischaemic colitis[Title/Abstract] OR splanchnic ischemia[Title/Abstract])) OR ("Mesenteric Ischemia"[Mesh] OR "Colitis, Ischemic"[Mesh])) AND (((((((((((((((((((biomarker[Title/Abstract] OR biological marker[Title/Abstract] OR biologic marker[Title/Abstract] OR "Biomarkers"[Mesh]) OR (intestinal Fatty Acid Binding Protein[Title/Abstract] OR intestinal fatty acid-binding protein[Title/Abstract] OR iFABP[Title/Abstract] OR I-FABP[Title/Abstract] OR intestinal ileal bile acid binding protein[Title/Abstract] OR I-BABP[Title/Abstract] OR "Fatty Acid-Binding Proteins"[Mesh] OR "Fatty Acid-Binding Proteins"[nm] OR "bile acid binding proteins"[Supplementary Concept])) OR (d-lactate[Title/Abstract] OR d lactate[Title/Abstract] OR l-lactate[Title/Abstract] OR l lactate[Title/Abstract] OR lactate[Title/Abstract] OR "Lactic Acid"[Mesh])) OR (alpha-GST[Title/Abstract] OR alpha-glutathione S-transferase[Title/Abstract] OR alpha glutathione s transferase[Title/Abstract] OR "glutathione S-transferase alpha"[Supplementary Concept] OR "Glutathione Transferase"[Mesh])) OR (CABA[Title/Abstract] OR cobalt-albumin binding assay[Title/Abstract] OR cobalt albumin binding assay[Title/Abstract] OR IMA[Title/Abstract] OR ischemia-modified albumin[Title/Abstract] OR ischemia modified albumin[Title/Abstract] OR ischaemia-modified albumin[Title/Abstract] OR ischaemia modified albumin[Title/Abstract] OR "ischemia-modified albumin"[Supplementary Concept])) OR (citrulline[Title/Abstract] OR "Citrulline"[Mesh])) OR (metabolic acidosis[Title/Abstract] OR metabolic acidoses[Title/Abstract] OR "Acidosis"[Mesh:NoExp])) OR (white blood cell count[Title/Abstract] OR leukocyte count[Title/Abstract] OR "Leukocyte Count"[Mesh:NoExp])) OR (C-reactive protein[Title/Abstract] OR CRP[Title/Abstract] OR "C-Reactive Protein"[Mesh])) OR (troponin[Title/Abstract] OR "Troponin"[Mesh] OR creatinine[Title/Abstract] OR "Creatinine"[Mesh])) OR (long non-coding RNA (lncRNA) H19[Title/Abstract] OR (lncRNA) H19[Title/Abstract] OR "RNA, Long Noncoding"[Mesh])) OR (HIF-1-alfa[Title/Abstract] OR Hypoxia-inducible factor 1-alpha[Title/Abstract] OR HIF-1α[Title/Abstract] OR HIF-1alpha[Title/Abstract] OR "Hypoxia-Inducible Factor 1, alpha Subunit"[Mesh])) OR (apelin[Title/Abstract] OR "Apelin"[Mesh])) OR (FGF-23[Title/Abstract] OR fibroblast growth factor 23[Title/Abstract] OR "Fibroblast Growth Factor-23"[Mesh])) OR (adropin OR human adropin protein OR "Enho protein, human"[Supplementary Concept])) OR (platelet-lymphocyte ratio OR "platelet to lymphocyte ratio" OR platelet/lymphocyte ratio OR platelet-to-lymphocyte ratio)) OR (neutrophil-lymphocyte ratio OR "neutrophil to lymphocyte ratio" OR neutrophil/lymphocyte ratio OR neutrophil-to-lymphocyte ratio)) OR (smooth muscle protein 22[Title/Abstract] OR SM22[Title/Abstract] OR "transgelin"[Supplementary Concept])) OR (D-dimer OR D-dimers OR "fibrin fragment D"[Supplementary Concept]))) AND ("Sensitivity and Specificity"[Mesh] OR sensitivity OR specificity OR "Predictive Value of Tests"[Mesh] OR predictive value OR positive predictive value OR PPV[Title/Abstract] OR negative predictive value OR NPV[Title/Abstract] OR "Likelihood Functions"[Mesh:NoExp] OR likelihood OR Receiver Operating Characteristics OR ROC[Title/Abstract] OR Area Under Curves OR AUC[Title/Abstract] OR AUROC[Title/Abstract] OR Diagnostic potential OR diagnostic predictor OR diagnostic accuracy OR diagnostic performance OR prognostic indicator OR prognostic role OR prognostic value OR diagnostic value OR false positive OR false negative OR true positive OR true negative OR efficient OR efficiency)) NOT (((("Letter" [Publication Type] OR "Editorial" [Publication Type] OR "Comment" [Publication Type] OR "Review" [Publication Type] OR "Case Reports" [Publication Type]) OR (letter[Title] OR comment*[Title])) OR (("Animals"[Mesh] NOT "Humans"[Mesh]) OR "Animal Experimentation"[Mesh] OR "Models, Animal"[Mesh])) OR ("Infant"[Mesh] OR "Child"[Mesh] OR infant OR newborn OR neonate OR baby OR child OR children OR kid OR kids))

**Scopus** 14 December 2022

| **Set** | **Query** |
| --- | --- |
| 7 | ( ( TITLE-ABS-KEY ( "mesenteric ishemia" OR " mesenteric ischemia" OR "mesenteric ischaemia" OR "mesentery artery ischaemia" OR "mesenteric artery ischaemia" OR "mesentery artery ischemia" OR "mesenteric artery ischemia" OR " mesenteric thrombosis" OR "mesenteric embolism" OR "mesenteric thrombus" OR "mesenteric embolus" OR "mesenteric thromboembolism" OR "bowel infarction" OR "mesenteric arterial thrombosis" OR "mesenteric arterial embolism" OR "mesenteric venous thrombosis" OR "nonocclusive mesenteric ischemia" OR "intestinal ischemia" OR "mesenteric infarction" OR "splanchnic ischemia" OR "bowel ischemia" OR "gut ischemia" OR "intestine vascular insufficiency" OR "mesenteric vascular insufficiency" OR "ischemic colitis" OR "ischaemic colitis" OR "intestinal gangrene" OR "bowel gangrene" OR "intestinal necrosis" OR "bowel necrosis" OR "abdominal compartment syndrome" OR "mesenteric embolus" OR "mesenteric thrombus" OR "mesenteric venous thrombosis" OR "non-occlusive intestinal ischemia" OR "nonocclusive intestinal ischemia" OR "non-occlusive intestinal ischaemia" OR "nonocclusive intestinal ischaemia" OR "non-occlusive mesenteric ischemia" OR "non-occlusive mesenteric ischaemia" OR "nonocclusive mesenteric ischaemia" ) ) AND ( ( TITLE-ABS-KEY ( biomarker OR "biological marker" OR "biologic marker" OR "intestinal fatty acid binding protein" OR "intestinal fatty acid-binding protein" OR ifabp OR i-fabp OR "intestinal ileal bile acid binding protein" OR i-babp OR "d-lactate" OR "d lactate" OR "l-lactate" OR "l lactate" OR lactate OR "alpha-gst" OR "alpha-glutathione s-transferase" OR "alpha glutathione s transferase" OR caba OR "cobalt-albumin binding assay" OR "cobalt albumin binding assay" OR ima OR "ischemia-modified albumin" OR "ischemia modified albumin" OR "ischaemia-modified albumin" OR "ischaemia modified albumin" OR "ischemia-modified albumin" OR citrulline ) ) OR ( TITLE-ABS-KEY ( "metabolic acidosis" OR "metabolic acidoses" OR "white blood cell count" OR "leukocyte count" OR "c-reactive protein" OR crp OR troponin OR creatinine OR "long non-coding rna lncrna h19" OR "lncrna h19" OR "hif-1-alfa" OR "hypoxia-inducible factor 1-alpha" OR hif-1α OR "hif-1 alpha" OR apelin OR "fgf-23" OR "fibroblast growth factor 23" OR adropin OR "human adropin protein" OR "enho protein, human" OR "platelet-lymphocyte ratio" OR "platelet to lymphocyte ratio" OR "platelet/lymphocyte ratio" OR "platelet-to-lymphocyte ratio" OR "neutrophil-lymphocyte ratio" OR "neutrophil to lymphocyte ratio" OR "neutrophil/lymphocyte ratio" OR "neutrophil-to-lymphocyte ratio" OR "smooth muscle protein 22" OR sm22 OR "transgelin" OR "d-dimer" OR "d-dimers" OR "fibrin fragment d" ) ) ) ) AND ( TITLE-ABS-KEY ( "sensitivity and specificity" OR sensitivity OR specificity OR "predictive value of tests" OR "predictive value" OR "positive predictive value" OR ppv OR "negative predictive value" OR npv OR likelihood OR "receiver operating characteristics" OR roc OR "area under curves" OR auc OR auroc OR "diagnostic potential" OR "diagnostic predictor" OR "diagnostic accuracy" OR "diagnostic performance" OR "prognostic indicator" OR "prognostic role" OR "prognostic value" OR "diagnostic value" OR "false positive" OR "false negative" OR "true positive" OR "true negative" OR efficient OR efficiency ) ) |
| 6 | ( TITLE-ABS-KEY ( "mesenteric ishemia" OR " mesenteric ischemia" OR "mesenteric ischaemia" OR "mesentery artery ischaemia" OR "mesenteric artery ischaemia" OR "mesentery artery ischemia" OR "mesenteric artery ischemia" OR " mesenteric thrombosis" OR "mesenteric embolism" OR "mesenteric thrombus" OR "mesenteric embolus" OR "mesenteric thromboembolism" OR "bowel infarction" OR "mesenteric arterial thrombosis" OR "mesenteric arterial embolism" OR "mesenteric venous thrombosis" OR "nonocclusive mesenteric ischemia" OR "intestinal ischemia" OR "mesenteric infarction" OR "splanchnic ischemia" OR "bowel ischemia" OR "gut ischemia" OR "intestine vascular insufficiency" OR "mesenteric vascular insufficiency" OR "ischemic colitis" OR "ischaemic colitis" OR "intestinal gangrene" OR "bowel gangrene" OR "intestinal necrosis" OR "bowel necrosis" OR "abdominal compartment syndrome" OR "mesenteric embolus" OR "mesenteric thrombus" OR "mesenteric venous thrombosis" OR "non-occlusive intestinal ischemia" OR "nonocclusive intestinal ischemia" OR "non-occlusive intestinal ischaemia" OR "nonocclusive intestinal ischaemia" OR "non-occlusive mesenteric ischemia" OR "non-occlusive mesenteric ischaemia" OR "nonocclusive mesenteric ischaemia" ) ) AND ( ( TITLE-ABS-KEY ( biomarker OR "biological marker" OR "biologic marker" OR "intestinal fatty acid binding protein" OR "intestinal fatty acid-binding protein" OR ifabp OR i-fabp OR "intestinal ileal bile acid binding protein" OR i-babp OR "d-lactate" OR "d lactate" OR "l-lactate" OR "l lactate" OR lactate OR "alpha-gst" OR "alpha-glutathione s-transferase" OR "alpha glutathione s transferase" OR caba OR "cobalt-albumin binding assay" OR "cobalt albumin binding assay" OR ima OR "ischemia-modified albumin" OR "ischemia modified albumin" OR "ischaemia-modified albumin" OR "ischaemia modified albumin" OR "ischemia-modified albumin" OR citrulline ) ) OR ( TITLE-ABS-KEY ( "metabolic acidosis" OR "metabolic acidoses" OR "white blood cell count" OR "leukocyte count" OR "c-reactive protein" OR crp OR troponin OR creatinine OR "long non-coding rna lncrna h19" OR "lncrna h19" OR "hif-1-alfa" OR "hypoxia-inducible factor 1-alpha" OR hif-1α OR "hif-1 alpha" OR apelin OR "fgf-23" OR "fibroblast growth factor 23" OR adropin OR "human adropin protein" OR "enho protein, human" OR "platelet-lymphocyte ratio" OR "platelet to lymphocyte ratio" OR "platelet/lymphocyte ratio" OR "platelet-to-lymphocyte ratio" OR "neutrophil-lymphocyte ratio" OR "neutrophil to lymphocyte ratio" OR "neutrophil/lymphocyte ratio" OR "neutrophil-to-lymphocyte ratio" OR "smooth muscle protein 22" OR sm22 OR "transgelin" OR "d-dimer" OR "d-dimers" OR "fibrin fragment d" ) ) ) |
| 5 | TITLE-ABS-KEY ( "mesenteric ishemia" OR " mesenteric ischemia" OR "mesenteric ischaemia" OR "mesentery artery ischaemia" OR "mesenteric artery ischaemia" OR "mesentery artery ischemia" OR "mesenteric artery ischemia" OR " mesenteric thrombosis" OR "mesenteric embolism" OR "mesenteric thrombus" OR "mesenteric embolus" OR "mesenteric thromboembolism" OR "bowel infarction" OR "mesenteric arterial thrombosis" OR "mesenteric arterial embolism" OR "mesenteric venous thrombosis" OR "nonocclusive mesenteric ischemia" OR "intestinal ischemia" OR "mesenteric infarction" OR "splanchnic ischemia" OR "bowel ischemia" OR "gut ischemia" OR "intestine vascular insufficiency" OR "mesenteric vascular insufficiency" OR "ischemic colitis" OR "ischaemic colitis" OR "intestinal gangrene" OR "bowel gangrene" OR "intestinal necrosis" OR "bowel necrosis" OR "abdominal compartment syndrome" OR "mesenteric embolus" OR "mesenteric thrombus" OR "mesenteric venous thrombosis" OR "non-occlusive intestinal ischemia" OR "nonocclusive intestinal ischemia" OR "non-occlusive intestinal ischaemia" OR "nonocclusive intestinal ischaemia" OR "non-occlusive mesenteric ischemia" OR "non-occlusive mesenteric ischaemia" OR "nonocclusive mesenteric ischaemia" ) |
| 4 | TITLE-ABS-KEY ( "sensitivity and specificity" OR sensitivity OR specificity OR "predictive value of tests" OR "predictive value" OR "positive predictive value" OR ppv OR "negative predictive value" OR npv OR likelihood OR "receiver operating characteristics" OR roc OR "area under curves" OR auc OR auroc OR "diagnostic potential" OR "diagnostic predictor" OR "diagnostic accuracy" OR "diagnostic performance" OR "prognostic indicator" OR "prognostic role" OR "prognostic value" OR "diagnostic value" OR "false positive" OR "false negative" OR "true positive" OR "true negative" OR efficient OR efficiency ) |
| 3 | ( TITLE-ABS-KEY ( biomarker OR "biological marker" OR "biologic marker" OR "intestinal fatty acid binding protein" OR "intestinal fatty acid-binding protein" OR ifabp OR i-fabp OR "intestinal ileal bile acid binding protein" OR i-babp OR "d-lactate" OR "d lactate" OR "l-lactate" OR "l lactate" OR lactate OR "alpha-gst" OR "alpha-glutathione s-transferase" OR "alpha glutathione s transferase" OR caba OR "cobalt-albumin binding assay" OR "cobalt albumin binding assay" OR ima OR "ischemia-modified albumin" OR "ischemia modified albumin" OR "ischaemia-modified albumin" OR "ischaemia modified albumin" OR "ischemia-modified albumin" OR citrulline ) ) OR ( TITLE-ABS-KEY ( "metabolic acidosis" OR "metabolic acidoses" OR "white blood cell count" OR "leukocyte count" OR "c-reactive protein" OR crp OR troponin OR creatinine OR "long non-coding rna lncrna h19" OR "lncrna h19" OR "hif-1-alfa" OR "hypoxia-inducible factor 1-alpha" OR hif-1α OR "hif-1 alpha" OR apelin OR "fgf-23" OR "fibroblast growth factor 23" OR adropin OR "human adropin protein" OR "enho protein, human" OR "platelet-lymphocyte ratio" OR "platelet to lymphocyte ratio" OR "platelet/lymphocyte ratio" OR "platelet-to-lymphocyte ratio" OR "neutrophil-lymphocyte ratio" OR "neutrophil to lymphocyte ratio" OR "neutrophil/lymphocyte ratio" OR "neutrophil-to-lymphocyte ratio" OR "smooth muscle protein 22" OR sm22 OR "transgelin" OR "d-dimer" OR "d-dimers" OR "fibrin fragment d" ) ) |
| 2 | TITLE-ABS-KEY ( "metabolic acidosis" OR "metabolic acidoses" OR "white blood cell count" OR "leukocyte count" OR "c-reactive protein" OR crp OR troponin OR creatinine OR "long non-coding rna lncrna h19" OR "lncrna h19" OR "hif-1-alfa" OR "hypoxia-inducible factor 1-alpha" OR hif-1α OR "hif-1 alpha" OR apelin OR "fgf-23" OR "fibroblast growth factor 23" OR adropin OR "human adropin protein" OR "enho protein, human" OR "platelet-lymphocyte ratio" OR "platelet to lymphocyte ratio" OR "platelet/lymphocyte ratio" OR "platelet-to-lymphocyte ratio" OR "neutrophil-lymphocyte ratio" OR "neutrophil to lymphocyte ratio" OR "neutrophil/lymphocyte ratio" OR "neutrophil-to-lymphocyte ratio" OR "smooth muscle protein 22" OR sm22 OR "transgelin" OR "d-dimer" OR "d-dimers" OR "fibrin fragment d" ) |
| 1 | TITLE-ABS-KEY ( biomarker OR "biological marker" OR "biologic marker" OR "intestinal fatty acid binding protein" OR "intestinal fatty acid-binding protein" OR ifabp OR i-fabp OR "intestinal ileal bile acid binding protein" OR i-babp OR "d-lactate" OR "d lactate" OR "l-lactate" OR "l lactate" OR lactate OR "alpha-gst" OR "alpha-glutathione s-transferase" OR "alpha glutathione s transferase" OR caba OR "cobalt-albumin binding assay" OR "cobalt albumin binding assay" OR ima OR "ischemia-modified albumin" OR "ischemia modified albumin" OR "ischaemia-modified albumin" OR "ischaemia modified albumin" OR "ischemia-modified albumin" OR citrulline ) |

**Web of Science** Core Collection 14 December 2022

(Topic) – title, abstract, author keywords, and Keywords Plus.

| **Set** | **Query** |
| --- | --- |
| 1 | intestinal OR intestine OR mesenteric OR mesentery OR bowel OR gut OR enteric OR enteral OR "small bowel" OR small-bowel OR "large bowel" OR large-bowel OR colon (Topic) |
| 2 | ischemia OR ischaemia OR ishemia OR ishaemia OR ishemic OR ishaemic OR ischemic OR ischaemic OR necrosis OR necrotic OR gangrene OR gangrenous OR infarction OR infarcted (Topic) |
| 3 | #1 AND #2 |
| 4 | abdominal compartment syndrome OR mesenteric arterial thrombosis OR mesenteric arterial embolism OR mesenteric embolus OR mesenteric thrombus OR mesenteric thromboembolism OR mesenteric venous thrombosis OR non-occlusive intestinal ischemia OR nonocclusive intestinal ischemia OR non-occlusive intestinal ischaemia OR nonocclusive intestinal ischaemia OR non-occlusive mesenteric ischemia OR nonocclusive mesenteric ischemia OR non-occlusive mesenteric ischaemia OR nonocclusive mesenteric ischaemia OR mesenteric vascular insufficiency OR vascular insufficiency of intestine OR intestinal vascular insuffuciency OR mesenteric infarction OR ischemic colitis OR ischaemic colitis OR splanchnic ischemia (Topic) |
| 5 | #3 OR #4 |
| 6 | biomarker OR biological marker OR biologic marker OR intestinal Fatty Acid Binding Protein OR intestinal fatty acid-binding protein OR iFABP OR I-FABP OR intestinal ileal bile acid binding protein OR I-BABP OR d-lactate OR d lactate OR l-lactate OR l lactate OR lactate OR alpha-GST OR alpha-glutathione S-transferase OR alpha glutathione s transferase OR CABA OR cobalt-albumin binding assay OR cobalt albumin binding assay OR IMA OR ischemia-modified albumin OR ischemia modified albumin OR ischaemia-modified albumin OR ischaemia modified albumin OR "ischemia-modified albumin" OR citrulline (Topic) |
| 7 | metabolic acidosis OR metabolic acidoses OR white blood cell count OR leukocyte count OR C-reactive protein OR CRP OR troponin OR creatinine OR long non-coding RNA lncRNA H19 OR lncRNA H19 OR HIF-1-alfa OR Hypoxia-inducible factor 1-alpha OR HIF-1α OR HIF-1alpha OR apelin OR FGF-23 OR fibroblast growth factor 23 OR adropin OR human adropin protein OR "Enho protein, human" OR platelet-lymphocyte ratio OR "platelet to lymphocyte ratio" OR platelet/lymphocyte ratio OR platelet-to-lymphocyte ratio OR neutrophil-lymphocyte ratio OR "neutrophil to lymphocyte ratio" OR neutrophil/lymphocyte ratio OR neutrophil-to-lymphocyte ratio OR smooth muscle protein 22 OR SM22 OR "transgelin" OR D-dimer OR D-dimers OR "fibrin fragment D" (Topic) |
| 8 | #6 OR #7 |
| 9 | #5 AND #8 |
| 10 | "Sensitivity and Specificity" OR sensitivity OR specificity OR "Predictive Value of Tests" OR predictive value OR positive predictive value OR PPV OR negative predictive value OR NPV OR likelihood OR Receiver Operating Characteristics OR ROC OR Area Under Curves OR AUC OR AUROC OR Diagnostic potential OR diagnostic predictor OR diagnostic accuracy OR diagnostic performance OR prognostic indicator OR prognostic role OR prognostic value OR diagnostic value OR false positive OR false negative OR true positive OR true negative OR efficient OR efficiency (Topic) |
| 11 | #9 AND #10 |

**Cochrane Library**  15 December 2022

| **Set** | **Query** |
| --- | --- |
| #1 | MeSH descriptor: [Mesenteric Ischemia] explode all trees |
| #2 | ("mesenteric ishemia" OR " mesenteric ischemia" OR "mesenteric ischaemia" OR "mesentery artery ischaemia" OR "mesenteric artery ischaemia" OR "mesentery artery ischemia" OR "mesenteric artery ischemia" OR " mesenteric thrombosis" OR "mesenteric embolism" OR "mesenteric thrombus" OR "mesenteric embolus" OR "mesenteric thromboembolism" OR "bowel infarction" OR "mesenteric arterial thrombosis" OR "mesenteric arterial embolism" OR "mesenteric venous thrombosis" OR "nonocclusive mesenteric ischemia" OR "intestinal ischemia" OR "mesenteric infarction" OR "splanchnic ischemia" OR "bowel ischemia" OR "gut ischemia" OR "intestine vascular insufficiency" OR "mesenteric vascular insufficiency" OR "ischemic colitis" OR "ischaemic colitis" OR "intestinal gangrene" OR "bowel gangrene" OR "intestinal necrosis" OR "bowel necrosis" OR "abdominal compartment syndrome" OR "mesenteric embolus" OR "mesenteric thrombus" OR "mesenteric venous thrombosis" OR "non-occlusive intestinal ischemia" OR "nonocclusive intestinal ischemia" OR "non-occlusive intestinal ischaemia" OR "nonocclusive intestinal ischaemia" OR "non-occlusive mesenteric ischemia" OR "non-occlusive mesenteric ischaemia" OR "nonocclusive mesenteric ischaemia"):ti,ab,kw (Word variations have been searched) |
| #3 | #1 OR #2 |
| #4 | MeSH descriptor: [Biomarkers] explode all trees |
| #5 | (biomarker OR "biological marker" OR "biologic marker" OR "intestinal fatty acid binding protein" OR "intestinal fatty acid-binding protein" OR ifabp OR i-fabp OR "intestinal ileal bile acid binding protein" OR i-babp OR "d-lactate" OR "d lactate" OR "l-lactate" OR "l lactate" OR lactate OR "alpha-gst" OR "alpha-glutathione s-transferase" OR "alpha glutathione s transferase" OR caba OR "cobalt-albumin binding assay" OR "cobalt albumin binding assay" OR ima OR "ischemia-modified albumin" OR "ischemia modified albumin" OR "ischaemia-modified albumin" OR "ischaemia modified albumin" OR "ischemia-modified albumin" OR citrulline OR "metabolic acidosis" OR "metabolic acidoses" OR "white blood cell count" OR "leukocyte count" OR "c-reactive protein" OR crp OR troponin OR creatinine OR "long non-coding rna lncrna h19" OR "lncrna h19" OR "hif-1-alfa" OR "hypoxia-inducible factor 1-alpha" OR hif-1Î± OR "hif-1 alpha" OR apelin OR "fgf-23" OR "fibroblast growth factor 23" OR adropin OR "human adropin protein" OR "enho protein, human" OR "platelet-lymphocyte ratio" OR "platelet to lymphocyte ratio" OR "platelet/lymphocyte ratio" OR "platelet-to-lymphocyte ratio" OR "neutrophil-lymphocyte ratio" OR "neutrophil to lymphocyte ratio" OR "neutrophil/lymphocyte ratio" OR "neutrophil-to-lymphocyte ratio" OR "smooth muscle protein 22" OR sm22 OR "transgelin" OR "d-dimer" OR "d-dimers" OR "fibrin fragment d"):ti,ab,kw (Word variations have been searched) |
| #6 | #4 OR #5 |
| #7 | #3 AND #6 |
| #8 | MeSH descriptor: [Sensitivity and Specificity] explode all trees |
| #9 | MeSH descriptor: [Predictive Value of Tests] explode all trees |
| #10 | MeSH descriptor: [Likelihood Functions] this term only |
| #11 | #8 OR #9 OR #10 |
| #12 | sensitivity OR specificity OR predictive value OR positive predictive value OR PPV OR "negative predictive value" OR NPV OR likelihood OR Receiver Operating Characteristics OR ROC OR Area Under Curves OR AUC OR AUROC OR Diagnostic potential OR diagnostic predictor OR diagnostic accuracy OR diagnostic performance OR prognostic indicator OR prognostic role OR prognostic value OR diagnostic value OR false positive OR false negative OR true positive OR true negative OR efficient OR efficiency |
| #13 | #11 OR #12 |
| #14 | #7 AND #13 |
