# Supplementary material for: Diagnostic accuracy of biomarkers to detect acute mesenteric ischaemia in adult patients: a systematic review and meta-analysis
Source: World J Emerg Surg. 2023 Sep 1;18:44. doi: 10.1186/s13017-023-00512-9 (PMC10474684; doi:10.1186/s13017-023-00512-9)
Supplement: Supplementary file 2 — Additional file 2: Table S1. Risk of bias assessment. [file 13017_2023_512_MOESM2_ESM.pdf]

Supplemental Table 1

First author, year of publication	Study design	Risk of bias				Applicability concerns		
		Patient selection	Index test	Reference test	Patient flow and timing	Patient selection	Index test	Reference test
Acosta 2001	Cohort	+	−	+	−	+	+	+
Acosta 2004	Cohort	+	−	+	+	+	+	+
Aktimur 2016	Case-control	−	+	+	−	+	+	+
Akyildiz 2009	Cohort	−	+	+	−	+	+	+
Arif 2016	Case-control	−	+	+	−	−	+	+
Arnalich 2010	Cohort	+	+	+	−	+	+	+
Bandea 2019	Cohort	−	+	+	−	−	+	+
Beng Fuh 2004	Cohort	+	−	+	−	+	+	+
Björnestadt 1993	Cohort	+	+	+	−	+	+	+
Block 2008	Cohort	+	−	+	+	+	+	+
Bogusevicius 2007	Cohort	−	+	+	−	−	+	+
Bourcier2022	Cohort	+	+	+	−	+	+	+
Brillantino 2018	Cohort	−	+	+	−	+	+	+
Calame 2021	Cohort	+	−	+	−	+	+	+
Chiu 2009	Cohort	+	−	+	−	+	+	+
Collange 2022	Case-control	−	+	+	−	+	+	+
Cosse 2013	Cohort	+	+	+	−	?	+	+
Cosse 2017	Cohort	−	+	+	−	+	+	+
Cronk 2006	Cohort	+	−	+	−	+	+	+
Dai 2018	Case-control	−	+	+	−	+	+	+
Degerli 2016	Case-control	−	+	−	−	+	+	+
Delaney 1999	Cohort	+	?	+	−	−	+	+
Durak 2022	Cohort	+	+	+	−	+	+	+
Edwards 2005	Case-control	−	−	+	−	+	+	+
Eyvaz 2021	Cohort	−	+	+	−	+	+	+

Ferrada 2017	Cohort	+	+	+	−	+	+	+
Fried 1991	Cohort	+	+	+	−	+	+	+
Gearhart 2003	Cohort	+	−	+	−	+	+	+
Groesdonk 2015	Cohort	+	+	+	−	+	+	+
Grotelueschen 2021	Cohort	+	−	+	−	+	+	+
Gün 2014	Cohort	−	+	+	−	+	+	+
Gunduz 2007	Case-control	−	+	+	−	+	+	+
Guzel 2013	Cohort	+	+	+	−	+	+	+
Hong 2017	Cohort	+	+	+	−	+	+	+
Hot 2016	Cohort	+	+	+	−	+	+	+
Huang 2018	Cohort	+	+	+	−	+	+	+
Icoz 2006	Cohort	+	?	+	−	+	+	+
Jancelewicz 2009	Cohort	+	−	+	−	−	+	+
Kanda 1996	Case-control	−	+	+	−	+	+	+
Kanda 2011	Cohort	+	+	+	−	+	+	+
Karadeniz 2020	Case-control	−	+	+	−	+	+	+
Kim 2020	Cohort	−	+	+	−	+	+	+
Kintu-Luwaga 2013	Cohort	−	?	+	−	+	+	+
Kisaoglu 2014	Case-control	−	?	+	−	+	+	+
Kittaka 2014	Cohort	+	+	+	−	+	+	+
Klingele 2015	Cohort	+	+	+	−	+	+	+
Koami 2015	Cohort	+	+	?	−	+	+	+
Kulu 2017	Cohort	−	?	+	−	+	+	+
Kurt 2022	Cohort	+	−	?	−	−	+	+
Lange 1997	Cohort	+	+	+	−	+	+	+
Li 2021	Cohort	+	+	+	−	−	+	+
Ludewig 2017	Case-control	?	+	+	−	+	+	+
Markogiannakis 2011	Case-control	−	+	+	−	?	+	+
Matsumoto 2014	Cohort	+	+	+	−	+	+	+
Matsumoto 2019	Cohort	+	+	+	−	+	+	+
Mothes 2021	Cohort	+	+	+	−	+	+	+
Murray 1994	Cohort	+	+	+	−	+	+	+
Nagata 2008	Cohort	+	+	+	−	+	+	+

Nilsson 2013	Cohort	+	+	+	−	+	+	+
Nuzzo 2017	Cohort	+	+	+	−	+	+	+
Nuzzo 2021	Cohort	+	+	+	−	+	+	+
Poeze 1998	Cohort	+	−	−	−	−	?	−
Polk 2008	Cohort	+	+	−	−	−	+	+
Sadot 2014	Cohort	−	+	+	−	−	+	+
Salim 2017	Cohort	+	+	+	−	+	+	+
Schoettler 2021	Case-control	−	+	+	−	−	+	+
Sekino 2017	Cohort	+	+	+	−	−	+	+
Sgourakis 2013	Cohort	−	+	+	−	−	+	+
Shi 2015	Cohort	+	+	+	−	+	+	+
Stroeder 2018	Cohort	+	+	+	−	+	+	+
Sutherland 2003	Cohort	−	+	+	−	+	+	+
Tanrikulu 2016	Cohort	+	+	+	−	+	+	+
Thuijls 2011	Cohort	+	+	+	−	+	+	+
Türkoglu 2015	Cohort	+	+	−	−	+	+	−
Uzun 2014	Cohort	+	+	?	−	+	+	?
van der Voort 2014	Cohort	−	+	+	−	−	+	+
Vermeulen Windsant 2012	Cohort	+	+	+	−	−	+	+
Wan 2019	Cohort	+	+	+	−	+	+	+
Woodford 2022	Cohort	+	+	+	−	−	+	+
Yamamoto 2005	Cohort	+	+	+	−	−	+	−
Yang 2014	Cohort	+	+	−	−	−	+	−
Zielinski 2016	Cohort	+	+	+	−	−	+	+
Zogheib 2018	Case-control	−	+	+	−	−	+	+
