# Supplementary material for: Diagnostic accuracy of biomarkers to detect acute mesenteric ischaemia in adult patients: a systematic review and meta-analysis
Source: World J Emerg Surg. 2023 Sep 1;18:44. doi: 10.1186/s13017-023-00512-9 (PMC10474684; doi:10.1186/s13017-023-00512-9)

Figure S1. Sensitivity (panel A) and specificity (panel B) of urinary intestinal fatty acid-binding protein (I-FABP) predicting AMI


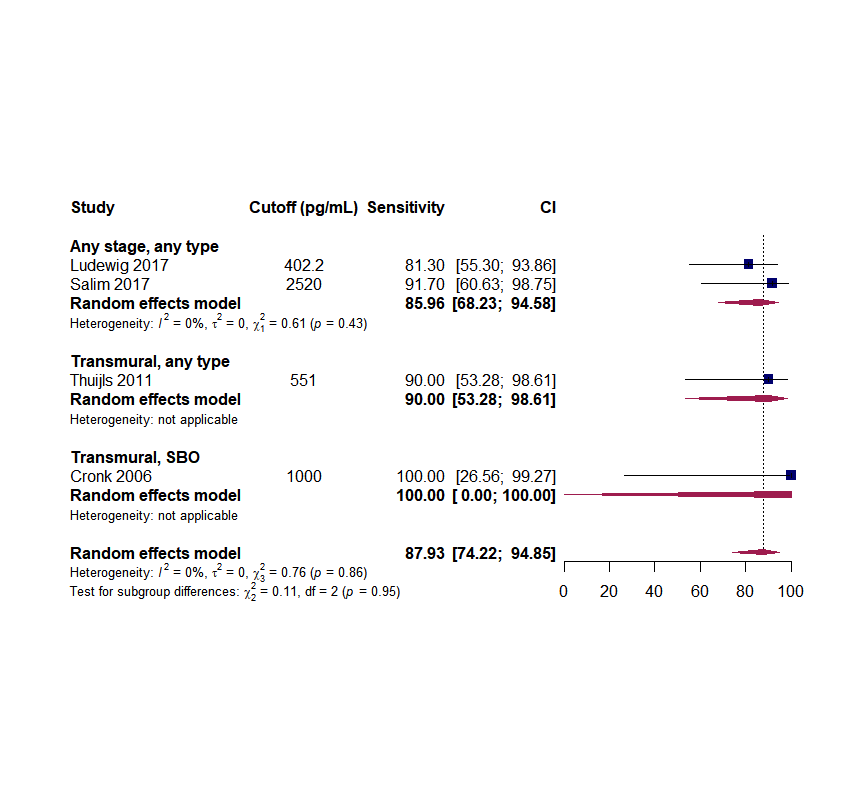

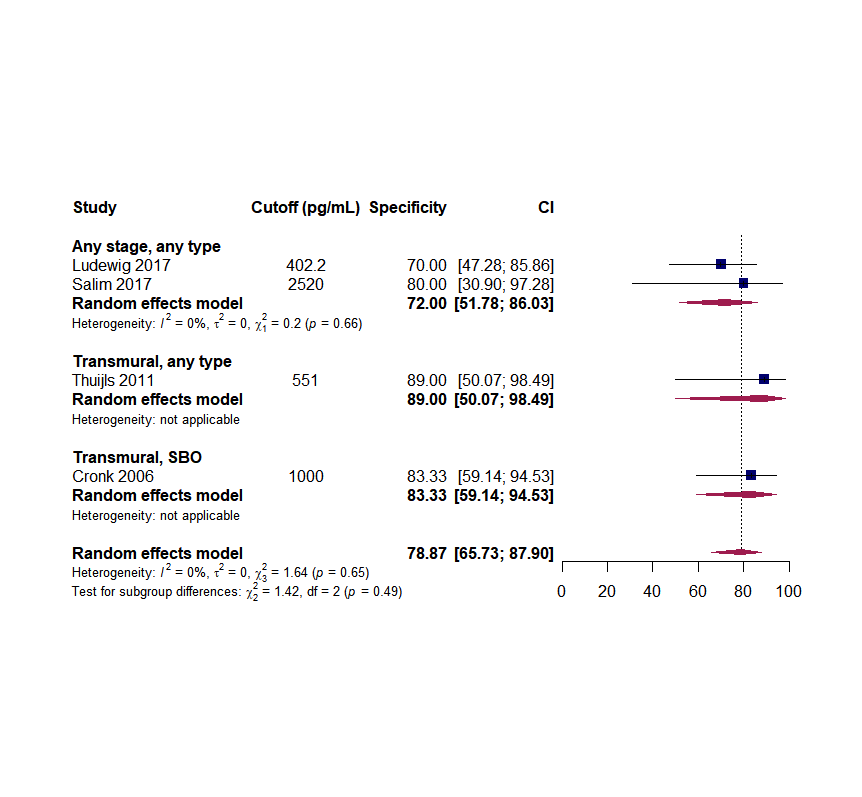


Figure S2. Sensitivity (panel A) and specificity (panel B) of serum C-reactive protein predicting AMI


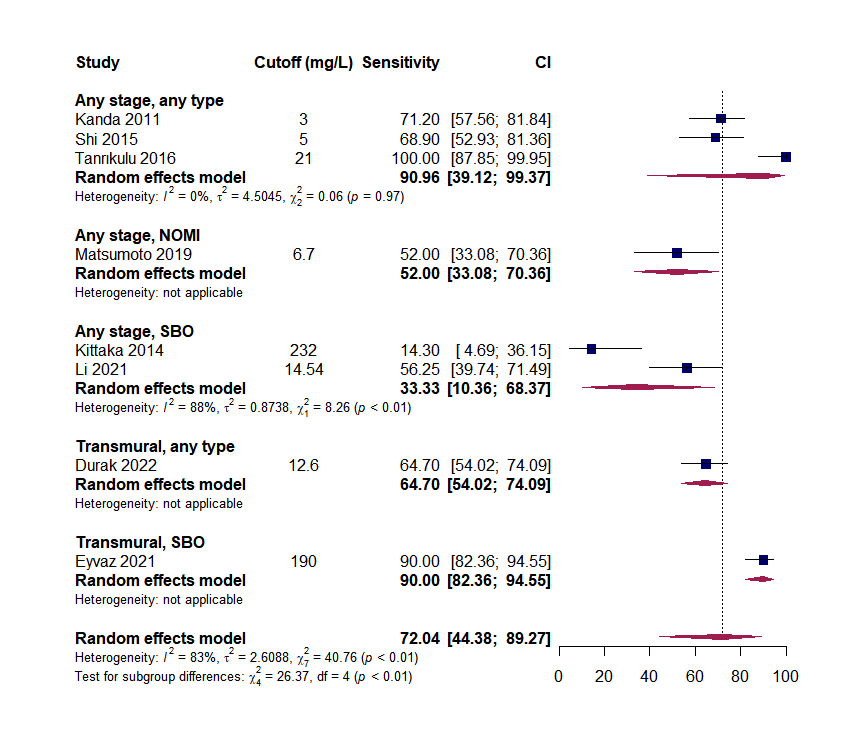

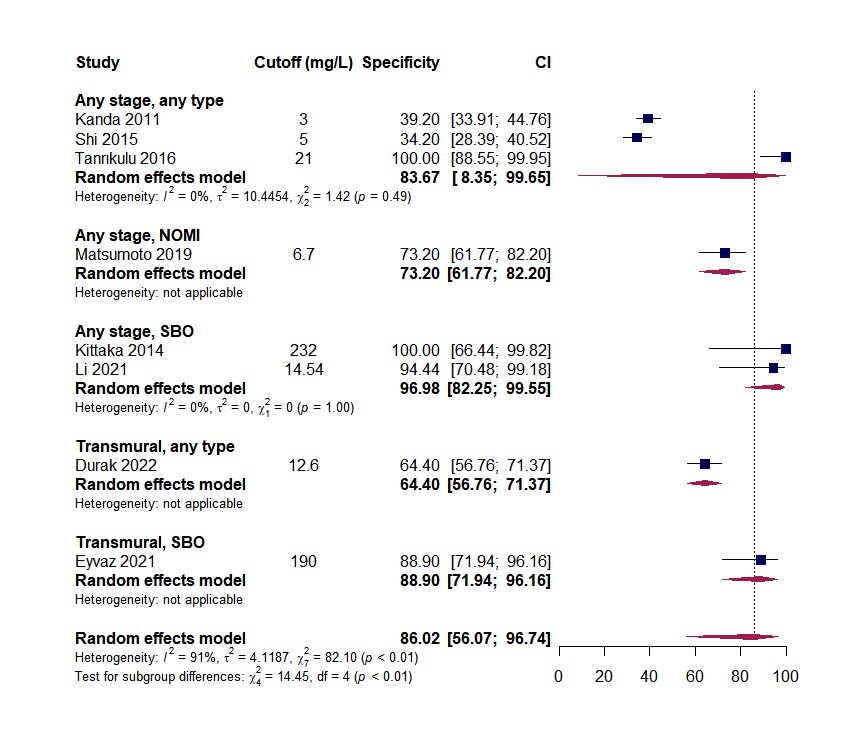


Figure S3. Sensitivity (panel A) and specificity (panel B) of serum procalcitonin predicting AMI


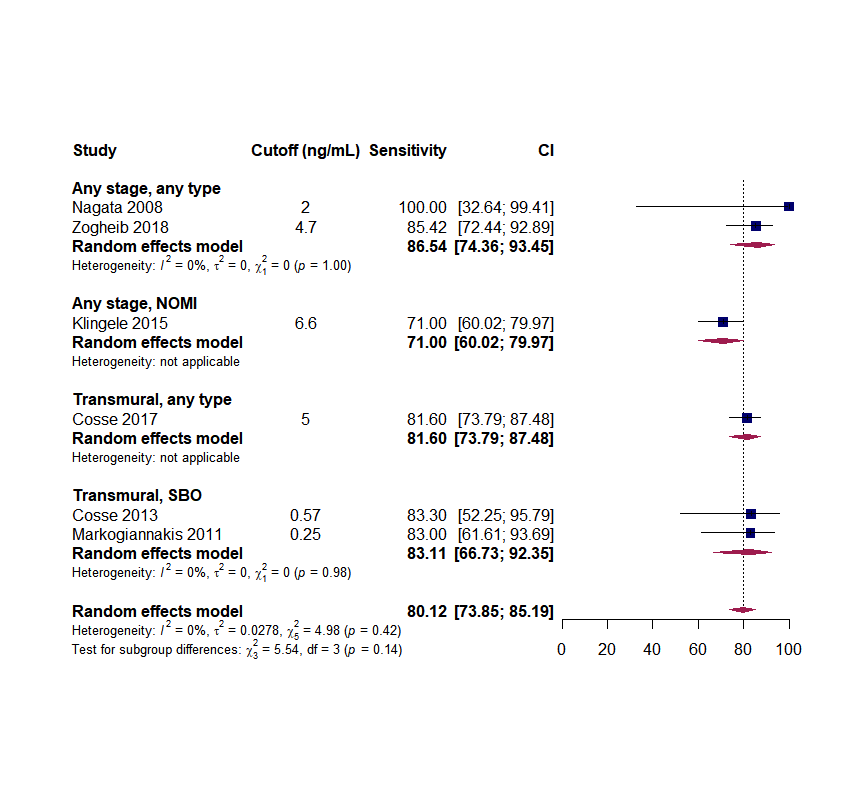

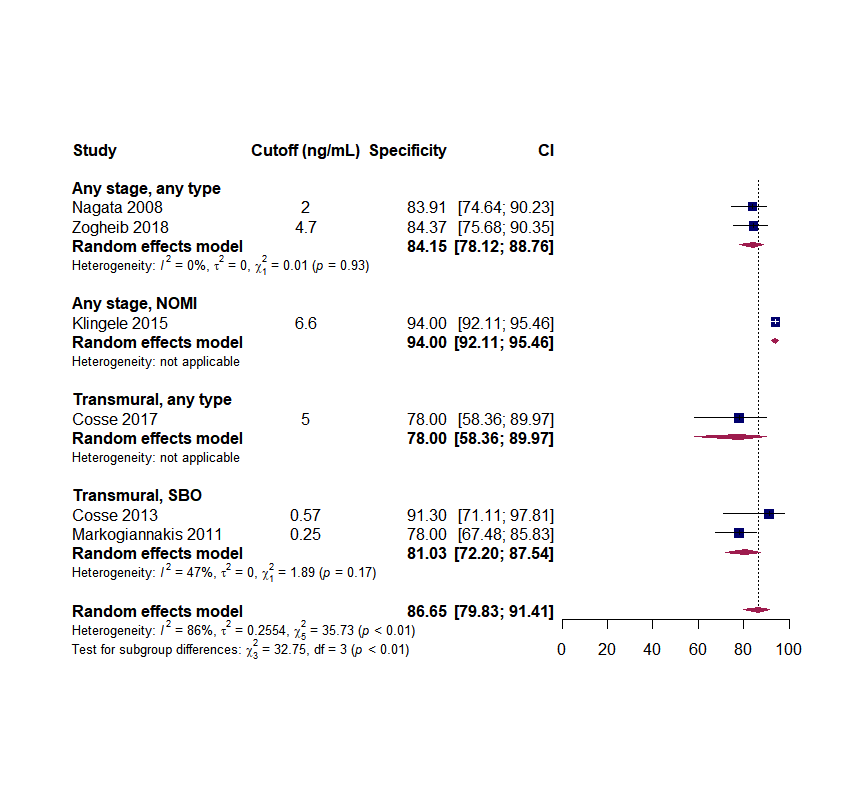


Figure S4. Sensitivity (panel A) and specificity (panel B) of serum interleukin-6 predicting AMI


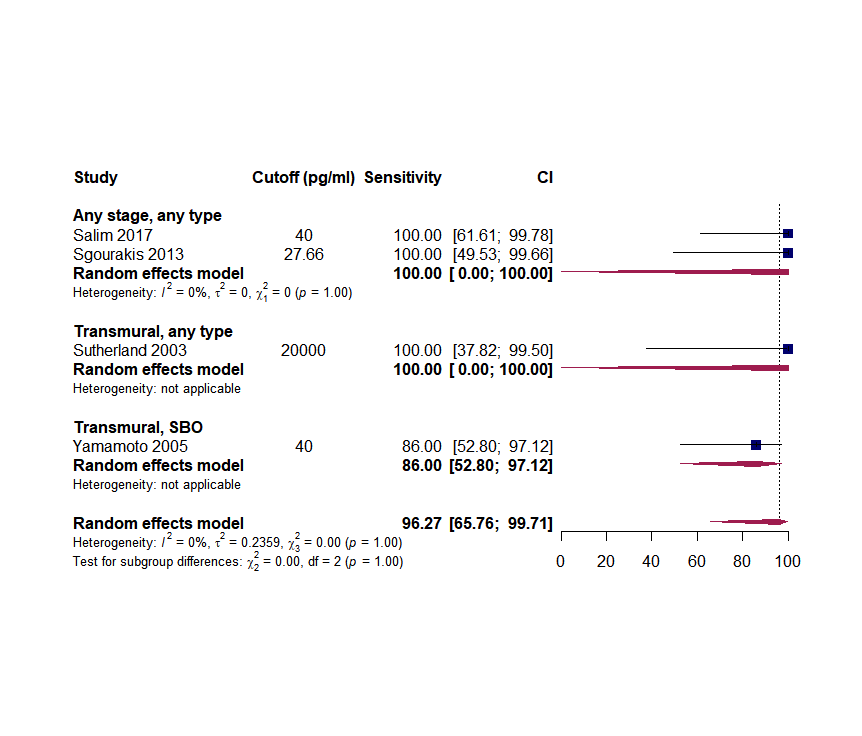

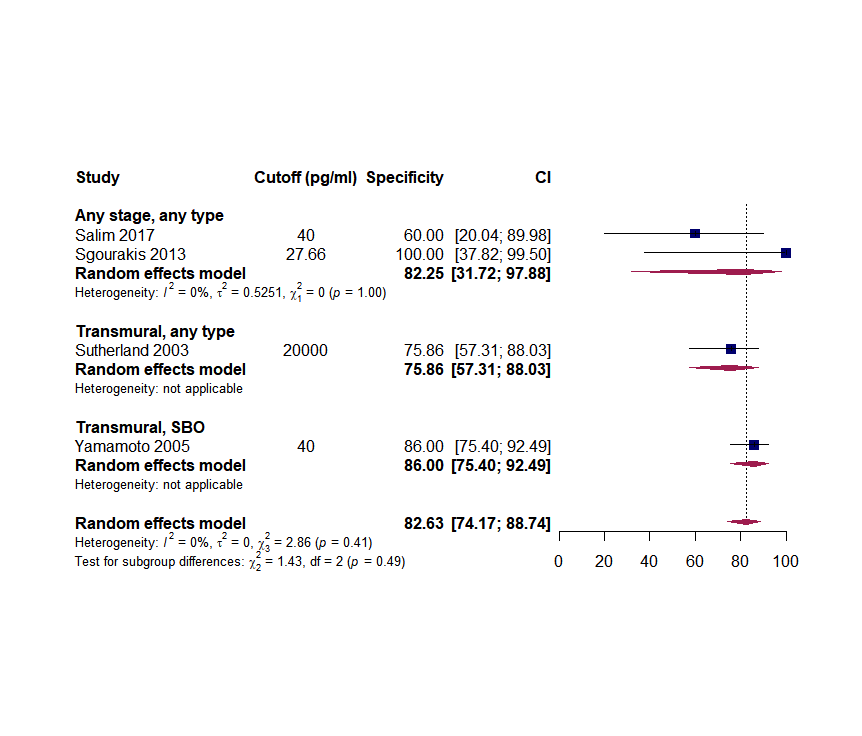


Figure S5. Sensitivity (panel A) and specificity (panel B) of serum alpha glutathione S transferase (alpha-GST) predicting AMI


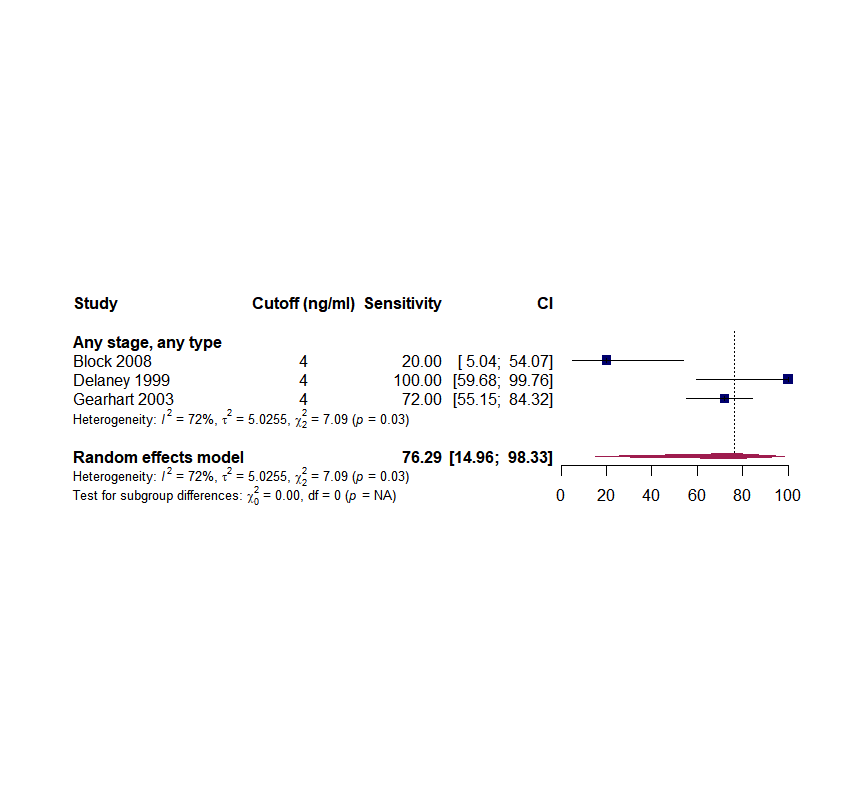

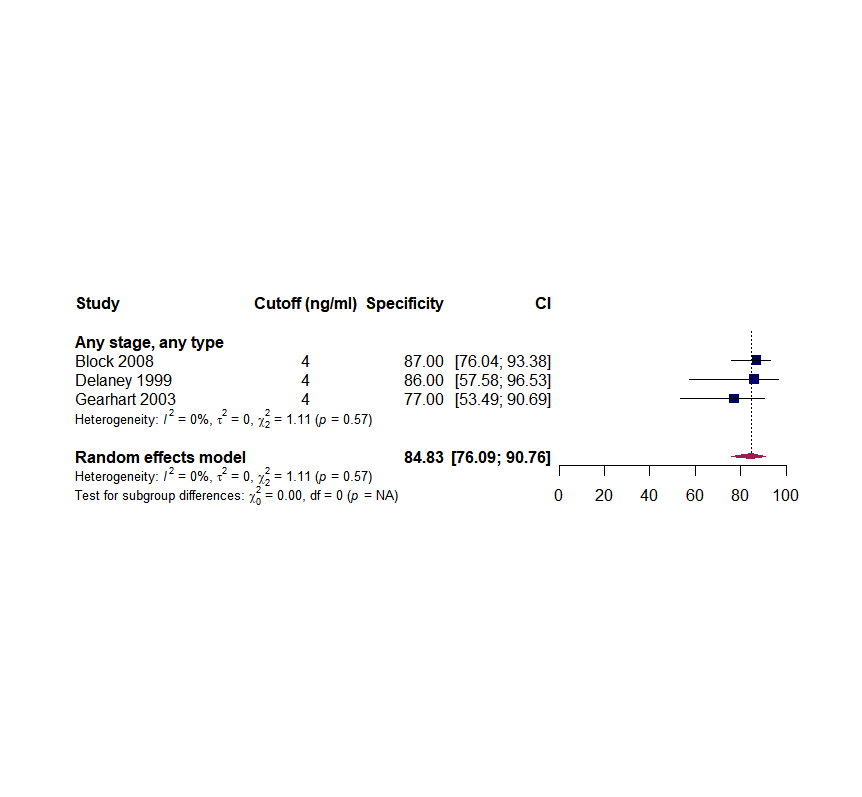


Figure S6. Sensitivity (panel A) and specificity (panel B) of serum citrulline predicting AMI


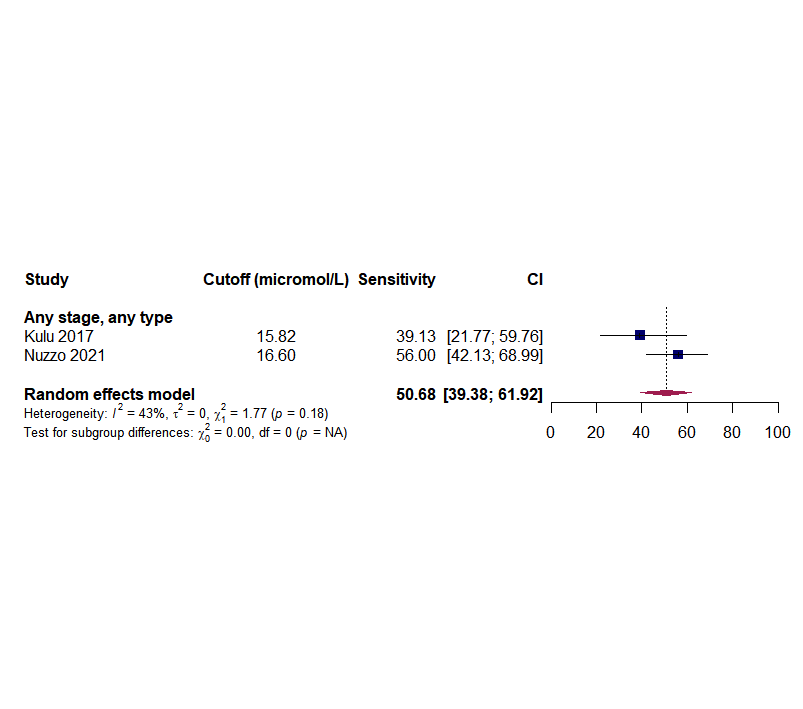

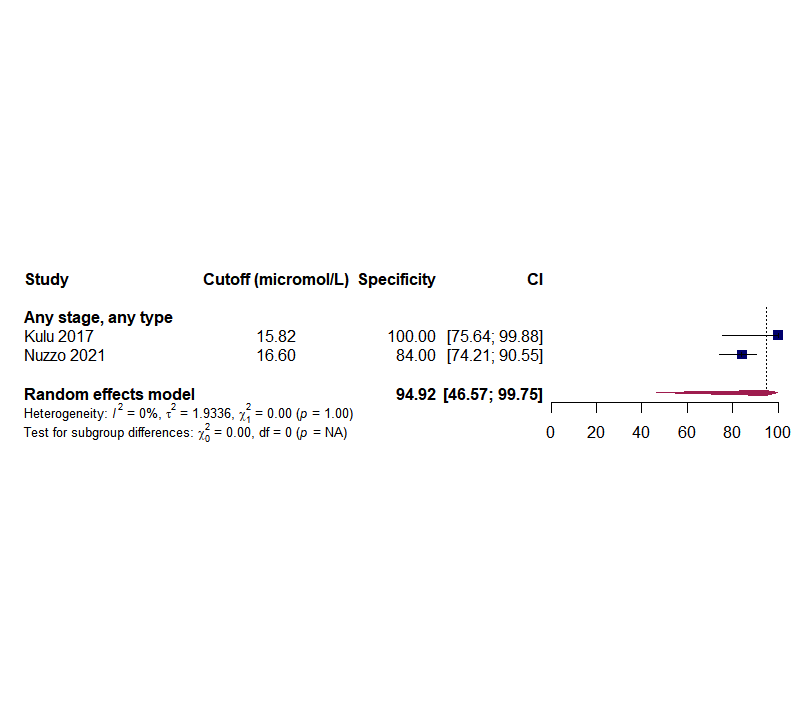


Figure S7. Sensitivity (panel A) and specificity (panel B) of serum ischaemia modified albumin (IMA) predicting AMI


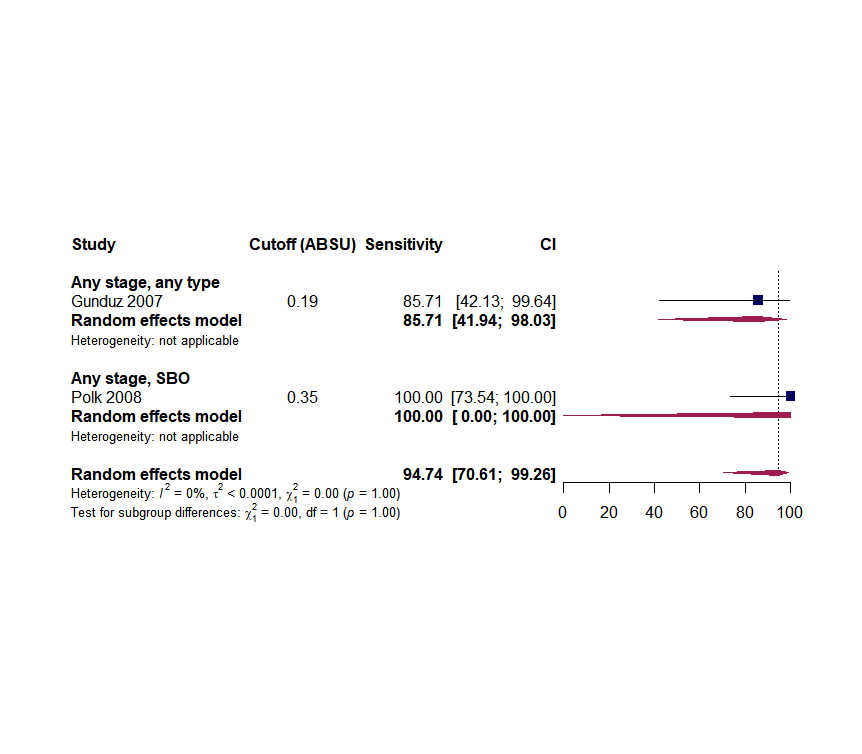

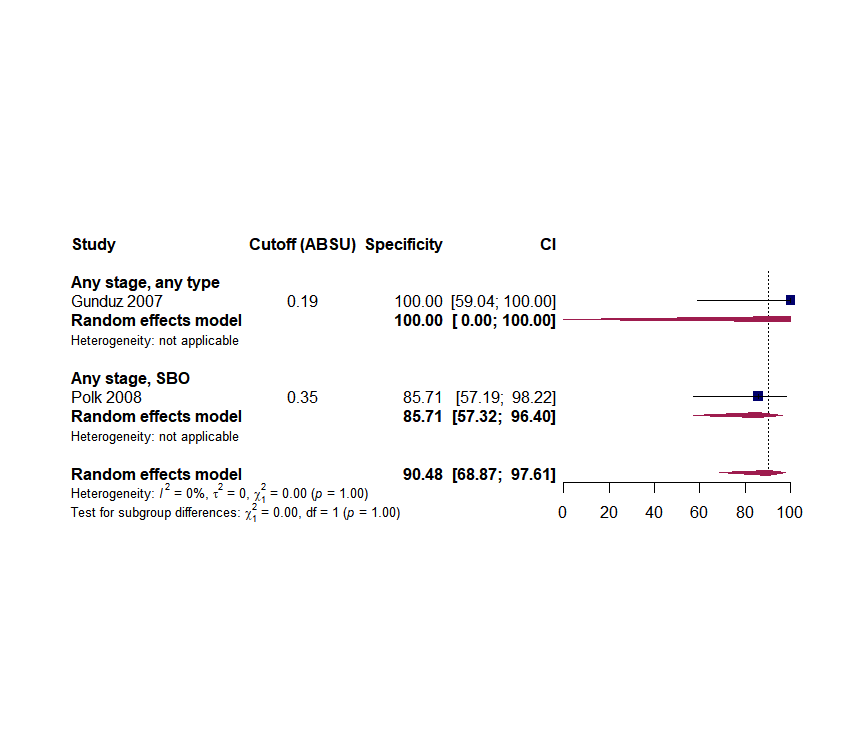


Legend: Gunduz 2007 included healthy volunteers as controls.

Figure S8. Sensitivity (panel A) and specificity (panel B) of mean platelet volume (MPV) predicting AMI


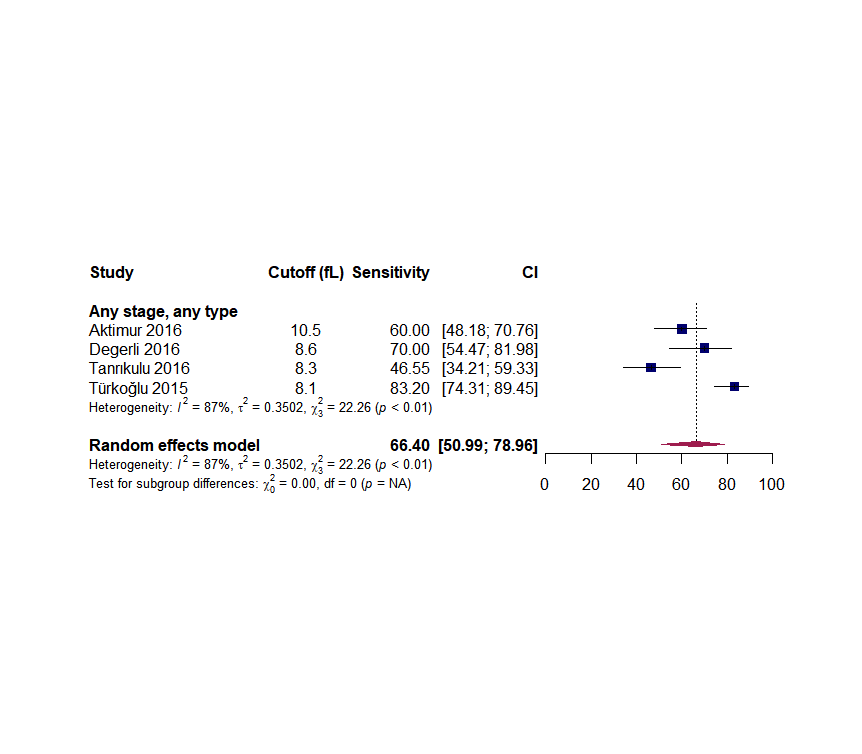

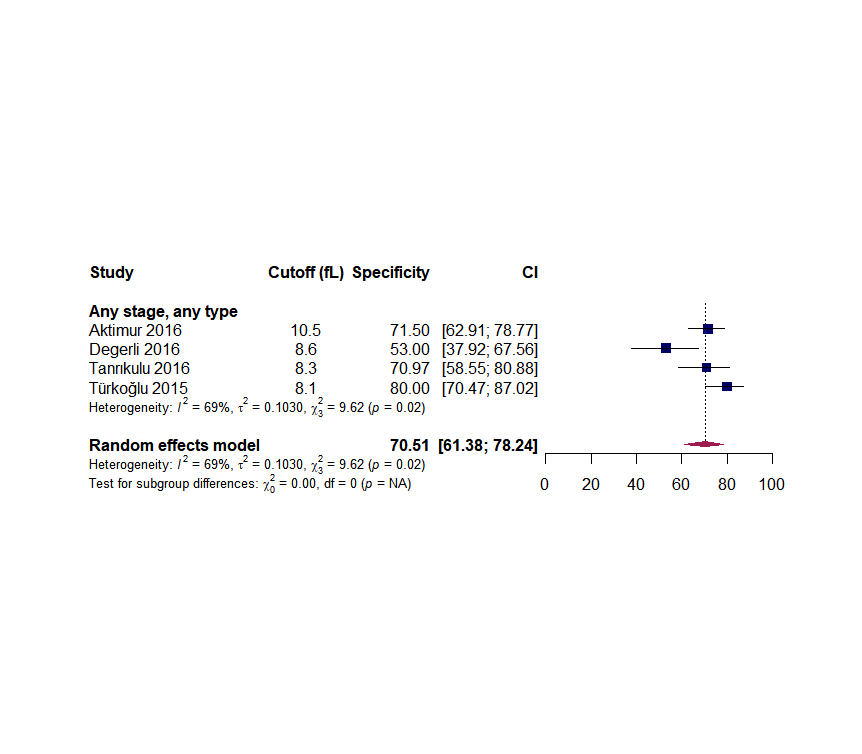


Legend: Degerli 2016 and Türkoglu 2015 included healthy volunteers as controls.

Figure S9. Sensitivity (panel A) and specificity (panel B) of red cell distribution width (RDW) predicting AMI


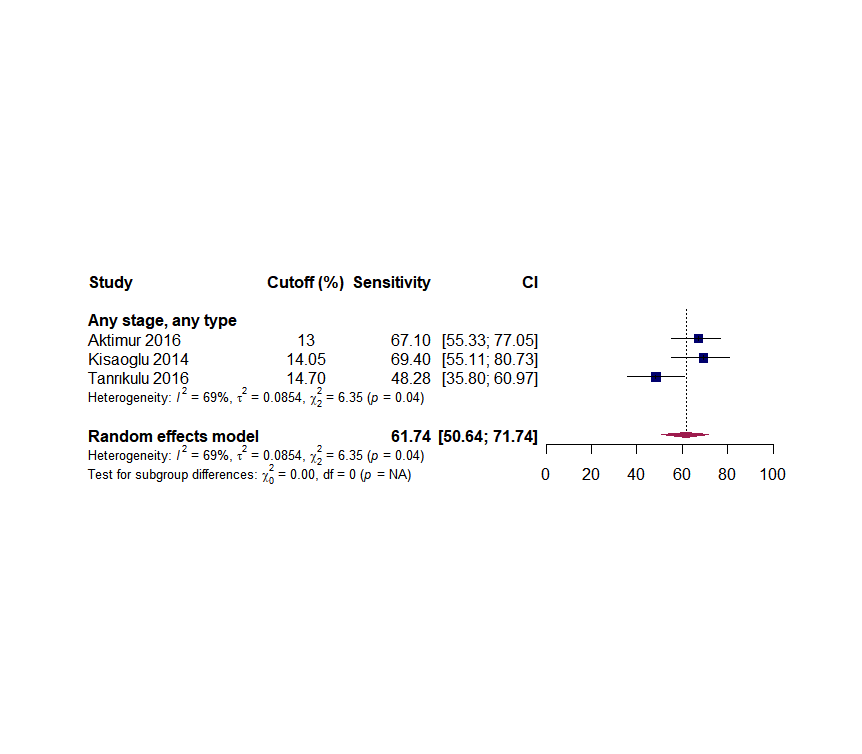

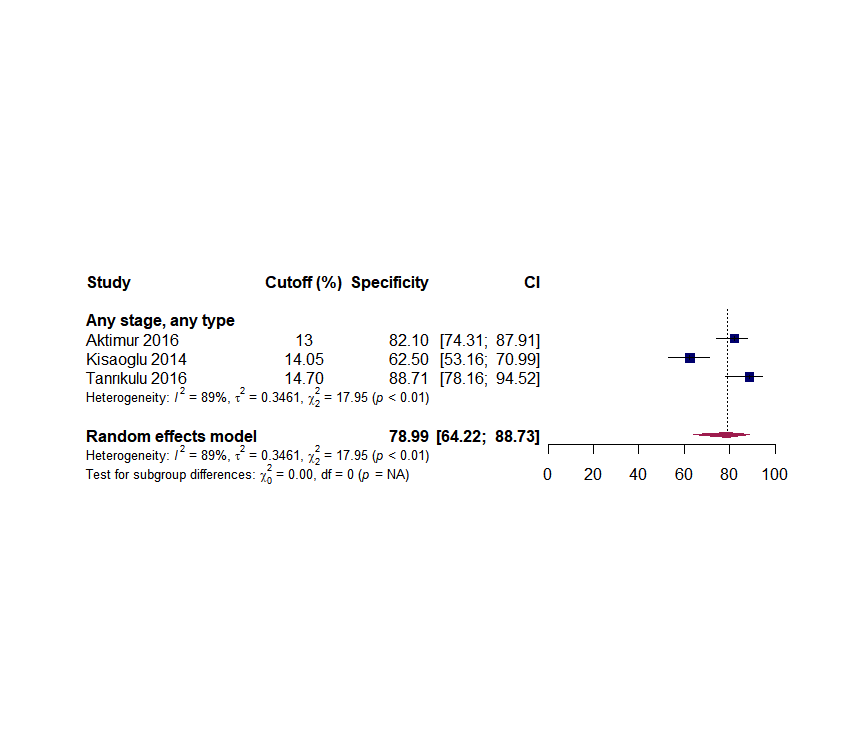


Figure S10. Sensitivity (panel A) and specificity (panel B) of neutrophil-lymphocyte ratio (NLR) predicting AMI


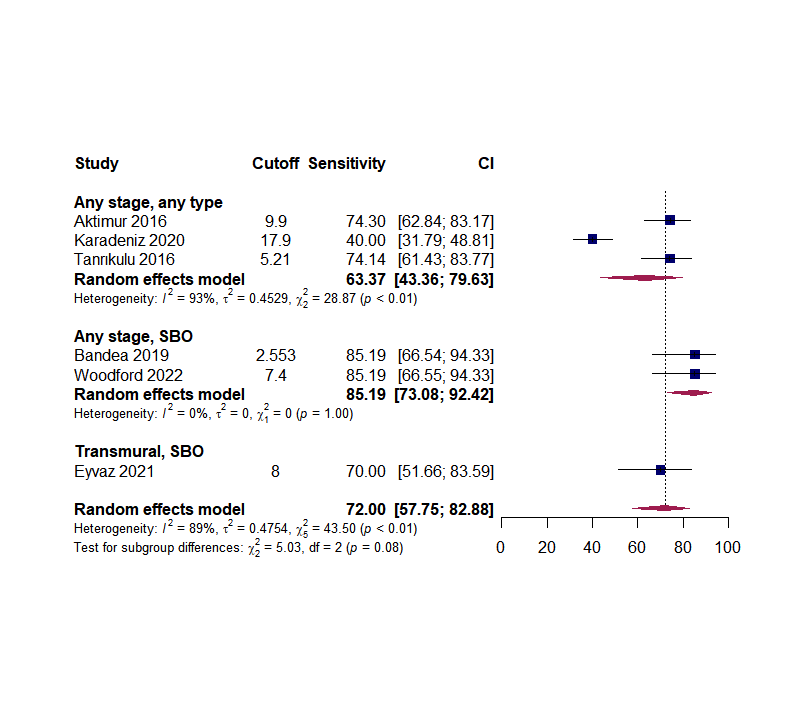

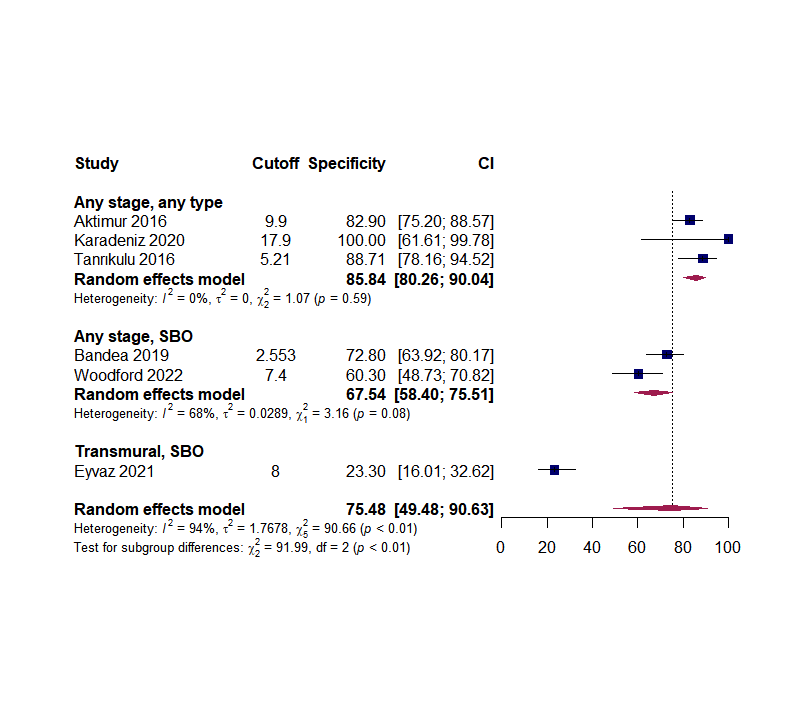


Figure S11. Sensitivity (panel A) and specificity (panel B) of platelet-lymphocyte ratio (PLR) predicting AMI


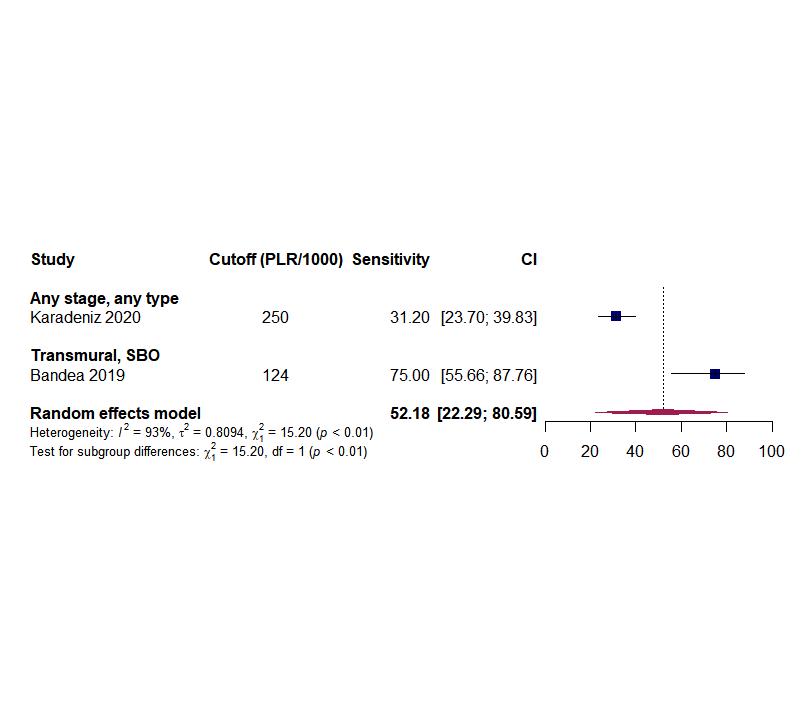

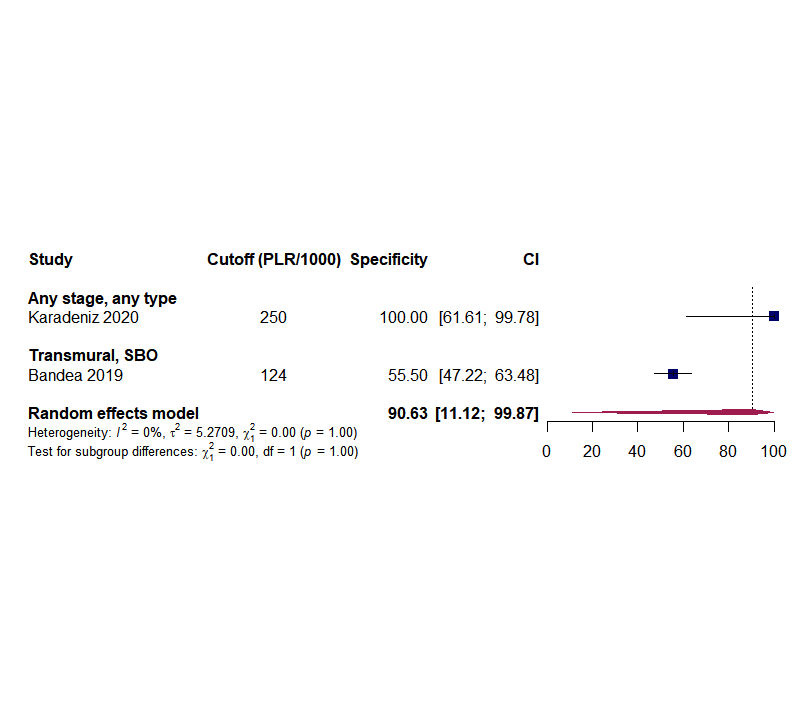


Figure S12. Sensitivity (panel A) and specificity (panel B) of serum lactate dehydrogenase (LDH) predicting AMI


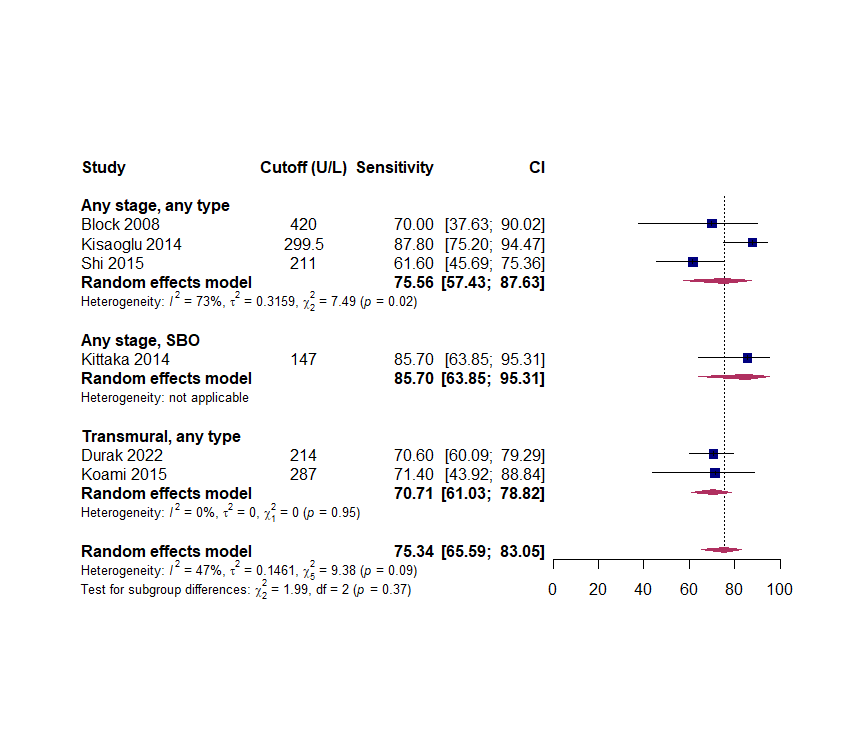

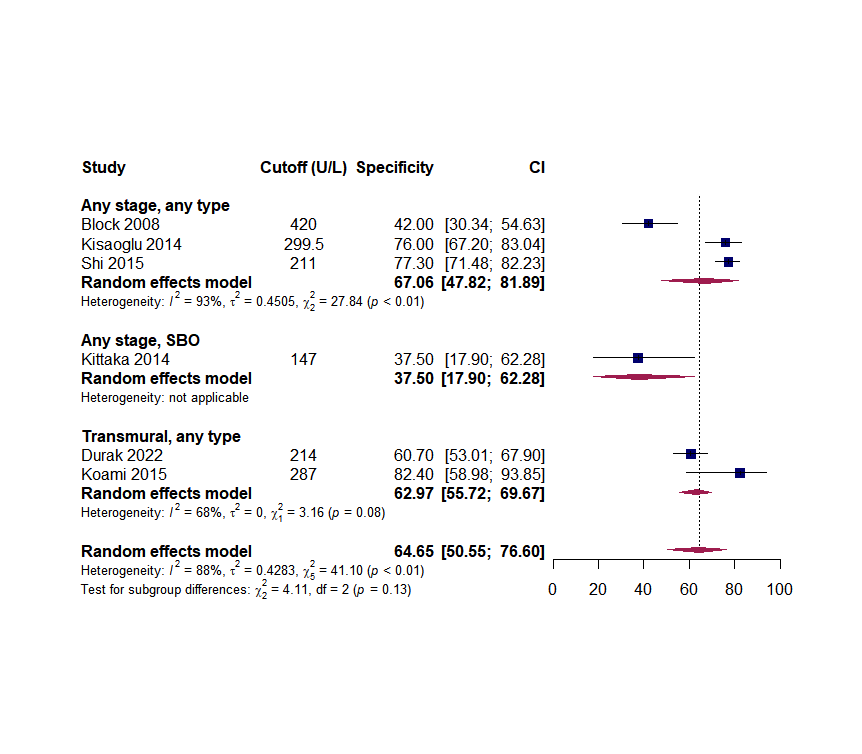


Figure S13. Sensitivity (panel A) and specificity (panel B) of serum D-lactate predicting AMI


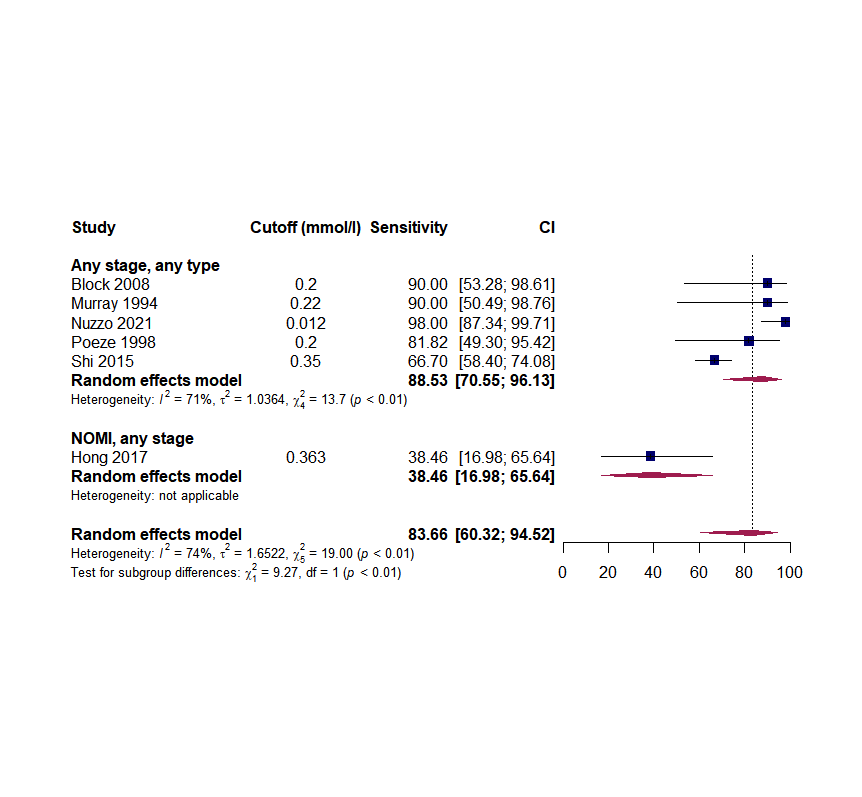

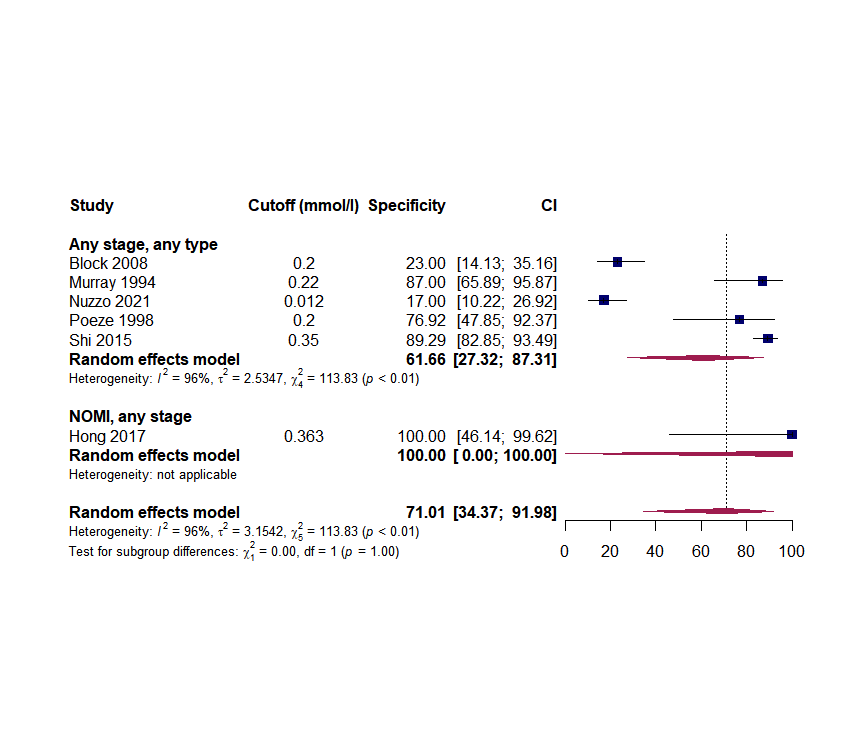


Figure S14. Sensitivity (panel A) and specificity (panel B) of serum pH predicting AMI


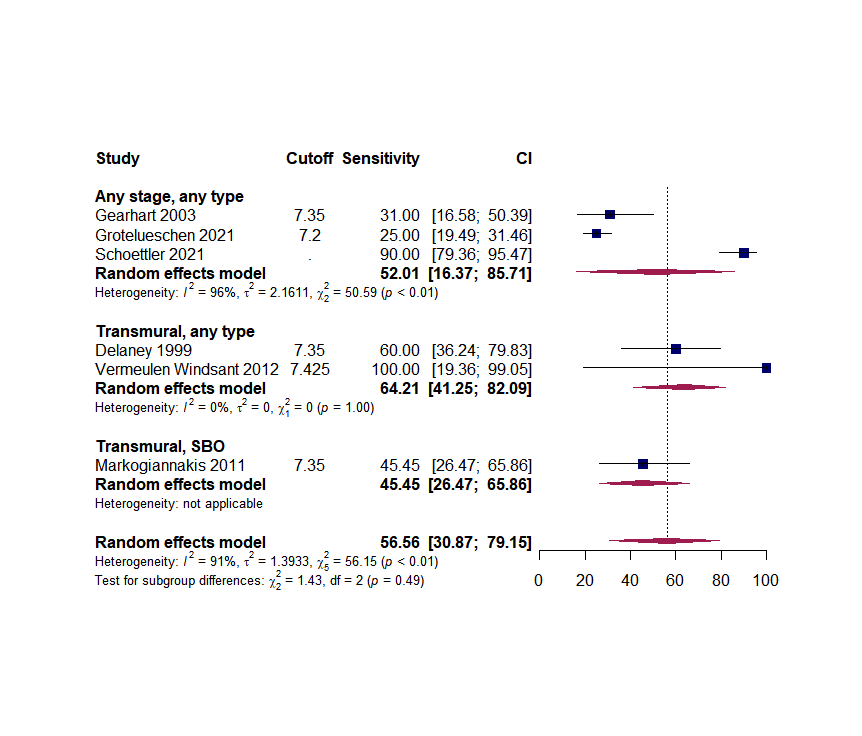

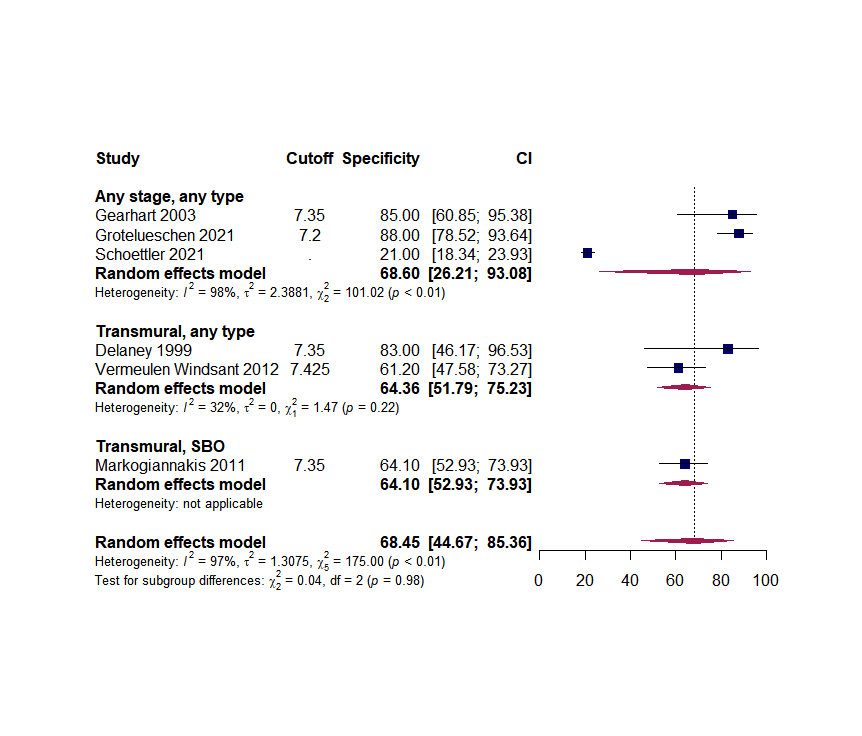


Legend:

Grotelueschen 2008: TP/FP/TN/FN numbers were used, reaching different sensitivity and specificity as reported in the original publication.

Figure S15. Sensitivity (panel A) and specificity (panel B) of serum bicarbonate predicting AMI


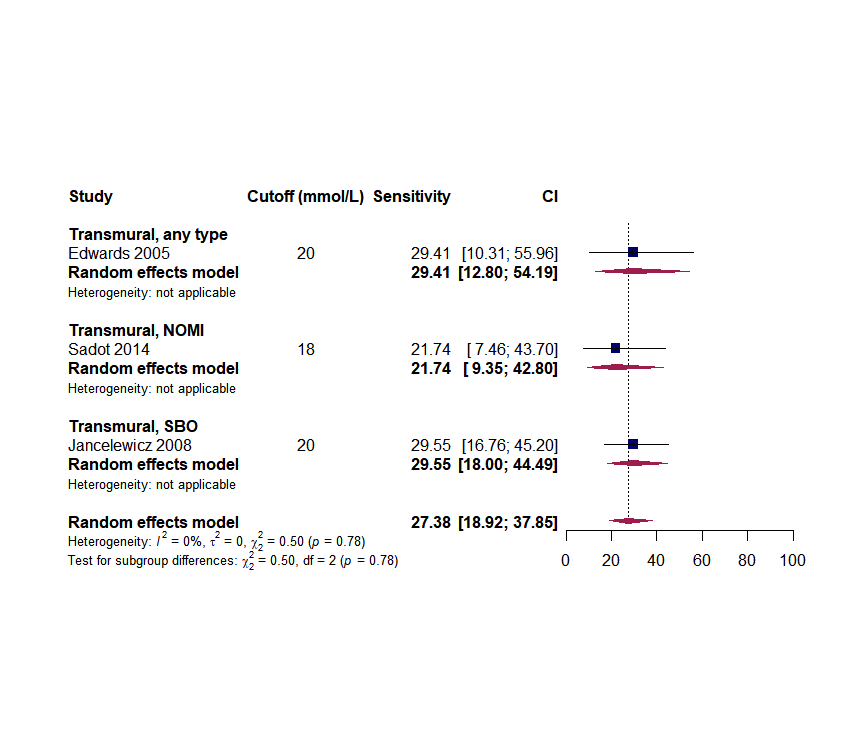

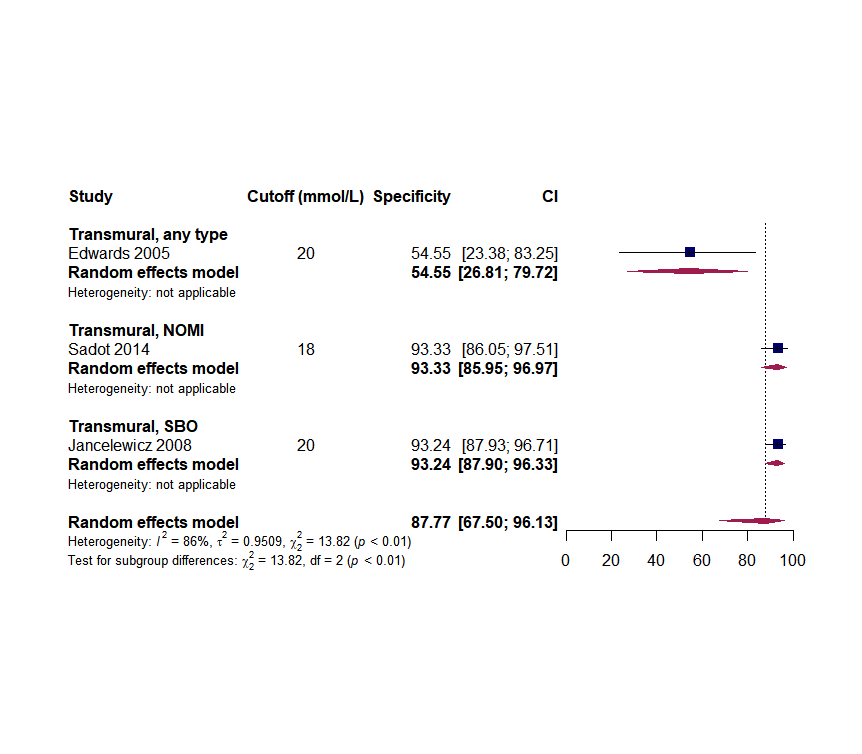

Supplement: Supplementary file 3 — Additional file 3: Figures S1-S15. Forest plots. [file 13017_2023_512_MOESM3_ESM.docx]
